# Comparative efficacy of leniolisib (CDZ173) versus standard of care on rates of respiratory tract infection and serum immunoglobulin M (IgM) levels among individuals with activated phosphoinositide 3-kinase delta (PI3Kδ) syndrome (APDS): an externally controlled study

**DOI:** 10.1093/cei/uxae107

**Published:** 2024-12-02

**Authors:** John Whalen, Anita Chandra, Sven Kracker, Stephan Ehl, Markus G Seidel, Ioana Gulas, Louis Dron, Russanthy Velummailum, Chenthila Nagamuthu, Sichen Liu, Joanne Tutein Nolthenius, Maria Elena Maccari

**Affiliations:** Pharming Group N.V., Leiden, The Netherlands; Department of Medicine, University of Cambridge, Cambridge, UK; Université Paris Cité, Imagine Institute, Laboratory of Human Lymphohematopoiesis, INSERM UMR 1163, F-7015, Paris, France; Institute for Immunodeficiency, Center for Chronic Immunodeficiency, Medical Center-University of Freiburg, Faculty of Medicine, University of Freiburg, Freiburg, Germany; Division of Pediatric Hematology Oncology, Department of Pediatrics and Adolescent Medicine, Medical University of Graz, Austria; Cytel Inc., Toronto, Ontario, Canada; Cytel Inc., Toronto, Ontario, Canada; Cytel Inc., Toronto, Ontario, Canada; Cytel Inc., Toronto, Ontario, Canada; Cytel Inc., Toronto, Ontario, Canada; Pharming Group N.V., Leiden, The Netherlands; Institute for Immunodeficiency, Center for Chronic Immunodeficiency, Medical Center-University of Freiburg, Faculty of Medicine, University of Freiburg, Freiburg, Germany; Division of Pediatric Hematology and Oncology, Department of Pediatrics and Adolescent Medicine, Medical Center-University of Freiburg, Faculty of Medicine, University of Freiburg, Freiburg, Germany

**Keywords:** APDS, ESID, immunoglobulin, infection, leniolisib, PI3Kδ

## Abstract

Leniolisib, an oral, targeted phosphoinositide 3-kinase delta (PI3Kδ) inhibitor, was well-tolerated and efficacious versus placebo in treating individuals with activated PI3Kδ syndrome (APDS), an ultra-rare inborn error of immunity (IEI), in a 12-week randomised controlled trial. However, longer-term comparative data versus standard of care are lacking. This externally controlled study compared the long-term effects of leniolisib on annual rate of respiratory tract infections and change in serum immunoglobulin M (IgM) levels versus current standard of care, using data from the leniolisib single-arm open-label extension study 2201E1 (NCT02859727) and the European Society for Immunodeficiencies (ESID) registry. The endpoints were chosen following feasibility assessment considering comparability and availability of data from both sources. Baseline characteristics between groups were balanced through inverse probability of treatment weighting. The leniolisib-treated group included 37 participants, with 62 and 49 participants in the control group for the respiratory tract infections and serum IgM analyses, respectively. Significant reductions in the annual rate of respiratory tract infections (rate ratio: 0.34; 95% confidence interval [CI]: 0.19, 0.59) and serum IgM levels (treatment effect: –1.09 g/L; 95% CI: –1.78, –0.39, *P* = 0.002) were observed in leniolisib-treated individuals versus standard of care. The results were consistent across all sensitivity analyses, regardless of censoring, baseline infection rate definition, missing data handling, or covariate selection. These novel data provide an extended comparison of leniolisib treatment versus standard of care, highlighting the potential for leniolisib to deliver long-term benefits by restoring immune system function and reducing infection rate, potentially reducing complications and treatment burden.

## Teaser

Longer-term data comparing the efficacy of leniolisib, an oral, targeted phosphoinositide 3-kinase delta (PI3Kδ) inhibitor, versus standard of care in individuals with activated PI3Kδ syndrome (APDS), are lacking. This externally controlled study compared the long-term effects of leniolisib on annual rate of respiratory tract infections and change in serum immunoglobulin M (IgM) levels versus current standard of care, using data from the leniolisib single-arm open-label extension study 2201E1 and the European Society for Immunodeficiencies (ESID) registry. Significant reductions in the annual rate of respiratory tract infections and serum IgM levels were observed in leniolisib-treated individuals versus standard of care, consistent across all sensitivity analyses, highlighting the potential for leniolisib to deliver long-term benefits.

## Graphical Abstract

**Graphical Abstract:**
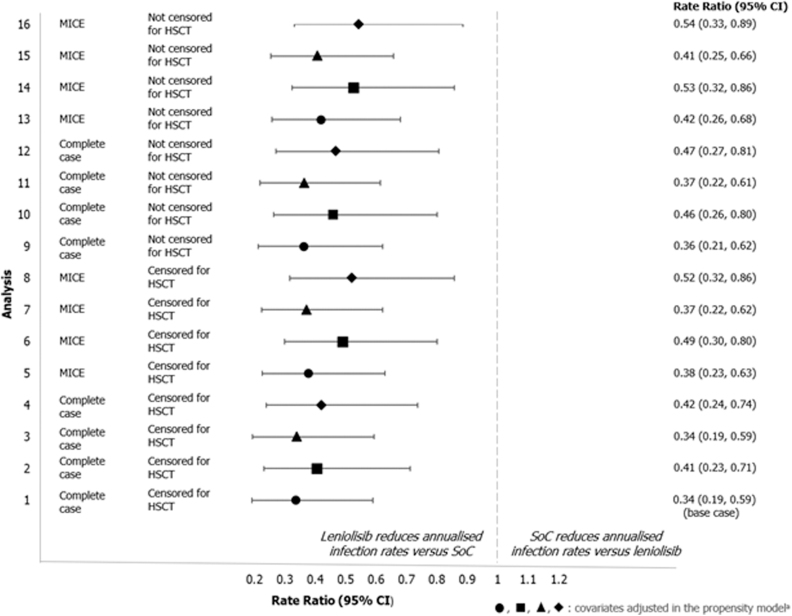

## Introduction

Activated phosphoinositide 3-kinase delta (PI3Kδ) syndrome (APDS) is an ultra-rare inborn error of immunity (IEI) caused by autosomal dominant mutations in the *PIK3CD* or *PIK3R1* genes, giving rise to APDS1 and APDS2, respectively [1–3]. By causing hyperactive signalling in the PI3Kδ pathway, APDS leads to the abnormal development, maturation, and proliferation of immune cells, resulting in combined immune deficiency and immune dysregulation [4–6]. Individuals with APDS present with higher-than-normal transitional B cells, with a reduced proportion of naïve B cells and fewer switched memory B cells [7–11]. Impaired immunoglobulin (Ig) class switching also results in reduced production of higher affinity antibodies such as IgG and IgA, and elevated levels of lower affinity IgM, impairing the ability to respond effectively to antigens [2, 10–14]. Furthermore, immunodeficiency in APDS is exacerbated by an accumulation of senescent and exhausted effector T cells, with fewer naïve and functional memory T cells [4, 5, 8, 11, 15–17].

Changes in immunophenotype and lymphocyte function lead to chronic and progressive symptoms in APDS [18, 19]. Almost all individuals with APDS experience early occurrence of severe recurrent respiratory infections, with onset of infections typically taking place in the first 10 years of life, affecting around 98% of patients by the age of 15 [5, 15, 20]. The combined frequency and severity of respiratory infections in APDS leads to chronic damage to the airways, resulting in bronchiectasis that is generally more severe than that observed in other IEIs [21]. In some instances, recurring severe cases of otitis media lead to permanent hearing loss [22]. People with APDS also experience a number of other manifestations, including lymphoproliferation (manifesting as lymphadenopathy, organomegaly and nodular lymphoid hyperplasia of the airways and gut), persistent herpesvirus infections, enteropathy, autoimmune cytopenia and an increased risk of malignancies, including lymphoma [5, 13, 15, 23].

For the majority of people, APDS management consists of a mix of immunosuppressive therapy and prophylaxis with immunoglobulin replacement therapy (IRT), which is indicated in patients with a primary immunodeficiency with impaired antibody production, alongside prophylactic antibiotics [5, 15, 23, 24]. Steroids and immunosuppressive therapies are frequently used for autoimmune or inflammatory complications [15, 24]. Surgeries, such as splenectomy or tonsillectomy, are also used to manage disease manifestations [23]. Haematopoietic stem cell transplantation (HSCT) has been used to cure immunological aspects of APDS, however, complications such as graft failure are reported after HSCT alongside associations of increased mortality [19, 25]. Therefore, the risks and benefits of HSCT must be carefully balanced and long-term outcomes remain to be fully evaluated.

Leniolisib, an oral, highly selective PI3Kδ inhibitor, is the first therapy approved for the treatment of ADPS that targets the underlying pathophysiology of the condition and is currently approved in the United States (US), the United Kingdom (UK), and Israel [26–29]. By reducing the production of phosphatidylinositol-3,4,5-triphosphate (PIP3) and inhibiting the subsequent recruitment and phosphorylation of a broad range of downstream messengers, leniolisib restores signalling homeostasis in the PI3Kδ pathway [26]. Long-term outcomes from the phase III open-label extension (OLE) have demonstrated that leniolisib was well-tolerated and maintained durable efficacy with up to 6 years of exposure, while also enhancing health-related quality of life (HRQoL) for patients [30, 31]. Patients treated with leniolisib experienced reductions in IgM levels versus patients treated with placebo in a 12-week phase III randomised controlled trial, and over a longer follow-up period in the OLE patients experienced a reduced number of infections, while 37% of patients who were receiving IRT at study entry reduced or discontinued IRT [31, 32]. For safety reasons, patients were required to discontinue most immunosuppressants prior to enrolling in the leniolisib trials, so there is a lack of data on the efficacy of leniolisib versus current standard of care [32].

The clinical development programme for leniolisib in adolescents and adults includes Study 2201 Part I, a single-arm, open-label, dose-finding study in six participants, and Part II (NCT02435173), a randomised, triple-blinded and placebo-controlled trial in 31 participants [32, 33]. Additionally, a single-arm OLE study (Study 2201E1, NCT02859727) has provided interim long-term data on the safety and efficacy of leniolisib with over five years of follow-up, with data collection ongoing [34, 35].

As Study 2201 Part II had a 12-week duration, and all participants in the OLE received leniolisib, long-term data comparing leniolisib with placebo or standard of care are lacking. Therefore, this study aimed to provide a long-term comparison by using an externally controlled design to compare the effects of leniolisib on the rate of respiratory tract infections and on serum IgM levels versus the current standard of care with symptomatic treatments in patients with APDS. Given that hyperactive PI3K signalling in APDS reduces class-switch recombination (resulting in excessive IgM production with reduced production of higher affinity antibodies such as IgG and IgA [2, 12–14]), in addition to increased transitional B cell numbers (which express IgM [2]), normalisation of IgM could indicate restoration of regulatory pathway balance (or homeostasis) and immune function. This would ultimately be seen through the return of endogenous IgG production.

The endpoints examined in this study (rate of respiratory tract infections and serum IgM level) were determined following feasibility assessment, with no long-term direct comparisons having been made between leniolisib and standard of care for these two endpoints before. Data from the leniolisib OLE study were compared to an external control sample population enrolled in the European Society for Immunodeficiencies (ESID) registry, the largest registry of individuals with primary immunodeficiencies worldwide [36].

## Materials and methods

The leniolisib OLE study recruited male and female participants aged 12–75 years inclusive with a documented APDS-associated genetic PI3Kδ mutation. Patients were enrolled from Study 2201 Part I (*n* = 6) and Study 2201 Part II (*n* = 29), regardless of their treatment allocation. Patients were also recruited from terminated clinical development programmes of other PI3Kδ inhibitors for APDS (*n* = 2) [37]. Full study designs and results for these trials have been reported previously [21, 32, 38].

The ESID registry is a prospective, observational, international, multicentre registry of individuals of all ages with an IEI, within which the APDS sub-registry collates extensive disease-specific data in participants with a validated diagnosis of APDS, consisting of initial retrospective documentation and yearly prospective follow-ups [21, 39]. Treatments received by individuals in the ESID-APDS registry include, but are not limited to, acute and prophylactic antibiotics, fungal prophylaxis, immunosuppressive treatments (including corticosteroids, rituximab, and mTOR inhibitors [e.g. sirolimus]), IRT, targeted treatments (e.g. PI3Kδ inhibitors), HSCT and varied surgical interventions. Outcomes following receipt of specific interventions were not reported in the dataset, and thus were not analysed.

In this study, participants from the OLE were considered the ‘treatment’ population and participants from the ESID-APDS registry represented the external ‘control’ population.

### Feasibility assessment

A feasibility assessment of potential effectiveness measures in the treatment and control populations, including the comparability of data available from the two data sources, was first performed to determine which endpoints were suitable for use within this study. The endpoints considered were the annual sum of respiratory infections, annualised change in serum IgM levels, naïve B cell counts, transitional B cell counts and proportion of patients achieving IRT freedom. Imaging endpoints, such as lymph node size and spleen size, were not considered as these data are not collected in the ESID registry.

Naïve B cell counts and transitional B cell counts were considered infeasible owing to the low sample size. Specifically, among patients in the ESID registry, only 22 and 14 patients had more than one entry with information on naïve B cell counts and transitional B cells, respectively. Meanwhile, although information on IRT use was repeatedly measured for the majority of patients in the ESID registry, date and duration of use, dose intensity and specific drug name data were not available in this dataset. Therefore, the annual sum of respiratory infections and the annualised change in serum IgM levels were chosen as the outcomes to be investigated in this study.

### Eligibility criteria

#### Treatment population

All OLE study participants who passed eligibility screening, the criteria for which have been previously published, were included in the analysis of respiratory infections [31, 32]. In the analysis of change in serum IgM levels, participants were included if they had at least two IgM values recorded over the course of treatment with leniolisib in the clinical programme. No patient had undergone HSCT during follow-up in the treatment population.

#### Control population

All participants in the ESID-APDS registry who consented to share their data with the OLE study sponsor were considered for inclusion in the study. However, patients were excluded from the respiratory infection analysis if they only had one recorded visit. Additional exclusion criteria applied for participants in the respiratory infections analysis were treatment with PI3Kδ inhibitors (leniolisib, nemiralisib, seletalisib or p110δ therapy) prior to or on the second visit, or receipt of HSCT prior to or on the second visit. Patients were excluded from the IgM analysis if they did not have two IgM tests ≤46 g/L recorded, two IgM tests prior to treatment with PI3Kδ inhibitors or had received HSCT prior to or on the date of their second IgM test [40].

The exclusion of patients who received HSCT was implemented to account for potential changes in data collection following receipt of HSCT in the ESID-APDS registry, either due to patients being followed up by different specialists post-HSCT or due to patients transitioning to different registries following HSCT, which may result in limited data collection post-HSCT. The impact of this exclusion criterion was explored in sensitivity analyses for both analyses.

### Endpoint definitions

#### Respiratory infections analysis

In the treatment population, respiratory infections were counted in 365-day periods starting from the date of the patient’s first dose of leniolisib in the OLE. The following infections, reported as adverse events in the study, were considered: otitis media, sinusitis, bronchitis, infective exacerbation of bronchiectasis, respiratory tract infection and pneumonia. Signs and symptoms of infection were monitored by investigators at each study visit and could be reported by participants or their healthcare practitioner outside of study visits.

In the control population, respiratory infections were counted in the interval between each registry visit, with a mean number of days between visits for the control population of 387.8 days (median: 349.0 days). All respiratory infections reported by investigators in the ESID-APDS registry were included: otitis media, sinusitis, chest infection and pneumonia.

#### Serum IgM analysis

For the treatment population, change in serum IgM values (g/L) was measured from the first IgM test recorded during leniolisib treatment as part of one of the clinical trials (Study 2201 Parts I/II, or the OLE for participants who were not part of Study 2201 or were initially assigned to placebo) to the last recorded IgM test. The last scheduled IgM test was on Day 252 of the OLE study. For participants from Study 2201 Parts I/II who had a treatment gap of ≥6 weeks before their first leniolisib dose in the OLE, the index test was defined as the first IgM test in the OLE. As the median interval between the first and last IgM tests recorded in the treatment population was 254 days, results were considered to be the rate of change per year, without adjustment.

In the control population, the annualised change in serum IgM values was measured from the first IgM test recorded in the ESID-APDS registry to the second IgM test, divided by time in years between the two tests. The rate of change was annualised due to the longer interval between IgM tests recorded in the ESID-APDS registry (3.1 years in the base case population). Alternative estimates were explored in sensitivity analyses.

### Follow-up

In all analyses, data were censored from the first occurrence of: participant withdrawal or death; receipt of HSCT (post-HSCT data were included in sensitivity analyses not censoring for HSCT) or a PI3Kδ inhibitor (control group only) and the end of the observation period. The last visit for infection reporting was 13^th^ September 2022 and 6^th^ February 2023 for the control and treatment populations, respectively. The last date of an IgM test was 8^th^ and 19^th^ September 2022 for the control and treatment population, respectively.

### Statistical analysis

Inverse probability of treatment weighting (IPTW) was used to minimise differences in the baseline characteristics reported for the treatment and control groups (Supplementary Methods 1.1). Propensity scores for each participant were calculated using a logistic regression model, with leniolisib assignment as the dependent variable. Parameters for inclusion within the propensity scores were determined through expert opinion.

In the respiratory infections analysis, covariates used as the independent variables included age at cohort entry, IRT use at baseline and baseline infection rate (Supplementary Table S1). Due to the small sample sizes and irregular number of intervals recorded within and across treatment groups, a generalised linear regression model using a Conway–Maxwell Poisson distributional family was used to evaluate the rate of respiratory infection by group (Supplementary Methods 1.2). Complete case analysis was used to exclude any participants with missing data.

In the serum IgM analysis, the covariates used were age at first IgM test, baseline serum IgM levels, APDS mutation status and sex (Supplementary Table S2). Propensity scores for each participant were then calculated using the fitted values from the logistic regression model, with average treatment effect in the overlap (ATO) weights calculated by IPTW. After assessing data for normality using the Shapiro–Wilk normality test, the annualised change in IgM indicated a non-normal distribution. Thus, a power of five transformation, determined via Box–Cox power transformation, was selected (Supplementary Fig. S1 and S2). A generalised linear model using complex survey design was used, and bootstrapping using a sampling replacement approach with 1,000 samples was used to calculate confidence intervals. Bootstrapping is a straightforward and commonly used technique to calculate confidence intervals for single data sets, requiring no assumptions of the sample distribution [41].

### Sensitivity analysis

To investigate the impact of methodological parameters of the study, a number of sensitivity analyses were conducted for the respiratory analysis and the serum IgM analysis.

#### Respiratory infections analysis

For the respiratory infections analysis, the impact of censoring follow-up at receipt of HSCT was explored by repeating the analyses without censoring participant follow-up at the point of receipt of HSCT. In these analyses, participants from both study groups were included regardless of whether HSCT was received after the index visit.

Alternative combinations of covariates for the propensity score model were examined, including a different definition of the baseline infection rate for the treatment group, and a more comprehensive list of clinically meaningful covariates, including baseline IgM level, sex and APDS mutation type (Supplementary Table S4).

In order to include patients with some missing data, the multiple imputation by chained equations (MICE) method was also explored as a procedure to recreate missing data [42]. The MICE procedure involved the construction of regression models for each variable, with missingness based on conditional distributions of the other variables in the data, across 50 imputed datasets (Supplementary Methods 1.5 and Supplementary Table S3).

Each of these parameters (including base case parameters) was combined to form a total of 15 sensitivity analyses.

#### Serum IgM analysis

In the serum IgM analysis, analyses were repeated without censoring participant follow-up at the point of receipt of HSCT. Participants in both study groups were included, unless the receipt of HSCT was before the first eligible IgM test.

To investigate the impact of outcome definitions, analyses of annualised IgM change in the control group were repeated by using the annualised change between the first and last IgM test, as well as the first IgM test and the subsequent test with the lowest IgM value during follow-up (Supplementary Table S5).

### Quantitative bias assessment

Non-randomised comparisons may be subject to bias, thus, a quantitative bias assessment (QBA) analysis was conducted to address any imbalances in the missingness of data and uncertainty in the capture of pertinent variables between the study populations. This analysis was conducted through a tipping-point approach, aiming to determine the impact of imbalanced covariates between the study groups, as well as the strength of unmeasured confounders that would reverse the conclusions of the analyses, measured as *E*-values.

### Results

#### Treatment population

In both analyses, 40 participants from the leniolisib clinical development programme and 134 participants from the ESID-APDS registry were assessed for inclusion. The final cohort for the analysis of respiratory infections included 37 treated and 62 control participants (Supplementary Fig. S3), while the final cohort for the analysis of IgM included 37 treated and 49 control participants (Supplementary Fig. S4; 37 and 52 participants, respectively, in the sensitivity analyses where patients were not censored for HSCT [Supplementary Fig. S5]). The majority of exclusions in the control population (*n* = 61 and *n* = 56 in the analyses of respiratory infections and serum IgM, respectively) resulted from participants having only one value for analysis (a single visit or IgM test).

#### Respiratory infections analysis

Baseline characteristics before and after IPTW are presented in Table 1, for the base case analysis. Balance was achieved in the age and sex covariates, however, receipt of IRT, serum IgM, the baseline infection rate, and APDS type remained imbalanced between the treatment population and control population representing patients receiving standard of care. Baseline characteristics before and after IPTW for all supplementary analyses are presented in the Supplementary Materials (Supplementary Table S6–S20).

**Table 1:** characteristics of participants with APDS from the treatment and control groups before and after IPTW for the base case respiratory infections analysis

Characteristic	Before weighting			After weighting^a^		
	Control	Treatment	SMD^b^	Control	Treatment	SMD^b^
Age at entry, years (median [IQR])	12.0 [7.0, 21.0]	21.0 [17.0, 29.0]	0.624	22.5 [11.3, 33.6]	20.5 [16.3, 29.0]	0.073
Sex (% male)	56.8	51.5	0.105	51.5	51.5	0.001
APDS subtype (% APDS2)	18.9	18.2	0.019	26.4	18.2	0.199
Infection rate (part I/II) (median [IQR]^c^)	0.0 [0.0, 0.005]	0.0 [0.0, 0.01]	0.027	0.004 [0.0, 0.009]	0.0 [0.0, 0.01]	0.207
Infection rate (183 days extension) (median [IQR])	0.0 [0.0, 0.006]	0.0 [0.0, 0.005]	0.469	0.004 [0.0, 0.009]	0.0 [0.0, 0.005]	0.643
IgM (log 10 + 1) (median [IQR])	0.5 [0.4, 0.6]	0.4 [0.2, 0.7]	0.255	0.5 [0.4, 0.6]	0.4 [0.2, 0.7]	0.178
Baseline IRT (% yes)	83.8	69.7	0.338	77.0	69.7	0.165

Results are reported for the base case analysis, in which missing data were handled via complete-case analysis. Age, IRT use and baseline infection rate (within Part I/II for the leniolisib group) were adjusted for in the propensity score model, and data was censored at the first occurrence of HSCT in the outcome model. After weighting, the effective sample size was *n* = 40 and *n* = 33 in the control and treatment group, respectively.

^a^Weights were truncated at the 5th and 95th percentile.

^b^Standardised mean difference ≥0.1 indicates imbalance.

^c^Timeframe for infection rate is equal to treatment time in trial 2201 Part I and II (84 and 85 days, respectively).

**Abbreviations:** APDS: activated phosphoinositide 3-kinase δ syndrome; IgM: immunoglobulin M; IRT: immunoglobulin replacement therapy; IQR: interquartile range; SMD: standardised mean difference.

Baseline characteristics that were not adjusted for using IPTW in the respiratory infection analysis for the treatment and control groups (all included participants, censored for HSCT) (Table 2) indicate the differences in baseline characteristics between the groups. In particular, the presence of lymphoproliferation was higher in the treatment population compared to the control group. Notably, the use of steroids was more common in the treatment population. Baseline characteristics for the cohort used in the sensitivity analyses (not censored for HSCT) are presented in Supplementary Table S21.

**Table 2:** clinical characteristics at or prior to baseline and during follow-up by group for the base case respiratory infections analysis (all included participants), after weighting

	At or prior to baseline			During follow-up		
	Control	Treatment	SMD^a^	Control	Treatment	SMD^a^
Presence of lymphoproliferation (% yes)	82.6	100	0.649	66.8	NA	
HSCT (% yes)^b^	0	0	<0.001	9.3	0	0.454
Respiratory tract infection (% yes)^c^	NA	NA		83.4	69.7	0.327
Any autoimmune cytopenia (% yes)	39.5	45.5	0.120	21.4	6.1^d^	0.456
Malignancy (% yes)	15.5	12.1	0.098	9.5	3.0	0.268
Concomitant medications^a^						
Antibiotics (% yes)	65.5	84.8	0.460	66.8	69.7	0.062
mTOR inhibitor^e^ (% yes)	36.9	24.2	0.278	43.6	0	1.244
Rituximab (% yes)	5.0	0	0.325	5.0	0	0.325
Steroids^f^ (% yes)	32.6	66.7	0.724	24.8	63.6	0.850

^a^Standardised mean difference ≥0.1 indicates imbalance.

^b^Participants who received HSCT prior to or on the date of their second visit (respiratory infections analysis) or second IgM test (IgM analysis) were excluded from analysis.

^c^Dates of infection were not available for the control population, thus, ‘baseline’ data were not available. Baseline infection rate for the treatment population was either based on Study 2201 Part I/II (if patients were enrolled in the trial), or based on infections that occurred in the first 183 days of the OLE study to be used as a proxy rate.

^d^Cytopenias during the leniolisib clinical studies were based on adverse event reports, and were due to any cause (so may not have been due to autoimmunity).

^e^Using definition based on any mention of mTOR throughout ESID variables.

^f^Steroid use in trial concomitant medication dataset included all drugs categorised as corticosteroids, glucocorticoids, steroid antibacterials or anticorticosteroids. **Abbreviations**: HSCT: haematopoietic stem cell transplant; mTOR: mammalian target of rapamycin; NA: not applicable; SMD: standardized mean difference.

**Figure 1:**
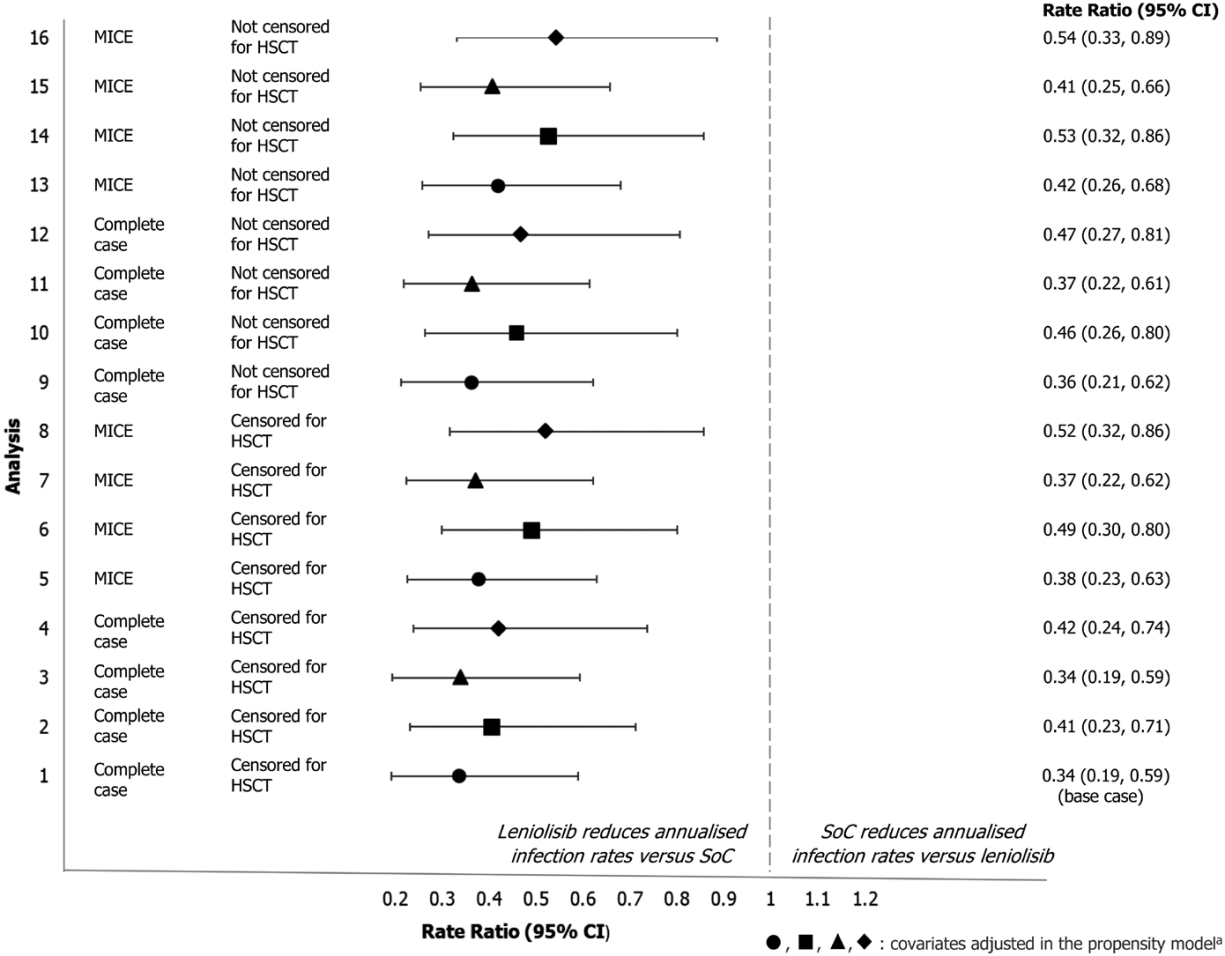
rate ratio (95%) CI for annualised infection rate for the treatment and control population across all sensitivity analyses. ^a^The symbols used in the figure indicate the covariates adjusted for in the propensity score model: ● age, IRT use, baseline infection rate (within Study 2201 Part I/II for treatment population), ■ age, IRT use, baseline infection rate (within first 183 days of OLE for treatment population), ▲ age, IRT use, baseline infection rate (within Study 2201 Part I/II for treatment population), IgM, sex, APDS type ◆ Age, IRT use, baseline infection rate (within first 183 days of OLE for treatment population), IgM, sex, APDS type Analysis 1 is the base case for the respiratory infection analysis; analyses 2–16 correspond to the sensitivity analyses described in Supplementary Table S6. **Abbreviations**: CI: confidence interval; HSCT: haematopoietic stem cell transplant; MICE: multiple imputation by chained equations; SoC: standard of care

The rate of respiratory infections was statistically significantly lower for trial participants who received leniolisib compared with the control population (Fig. 1), with estimated annualised rates of infection (95% confidence interval [CI]) of 0.45 (0.29, 0.70) and 1.34 (0.89, 2.00) per year, respectively. The rate ratio for annualised rates of respiratory infection (95% CI) was 0.34 (0.19, 0.59) for the treatment vs control population.

The results were consistent in all sensitivity analyses (Fig. 1) regardless of missing data handling, covariate selection, censoring at HSCT, or definition of the baseline infection rate, showing a consistent and statistically significant reduction of 46–66% in annual infection rates for participants treated with leniolisib (Supplementary Table S22–25). In the QBA analysis, rate ratios remained significantly in favour of leniolisib after adjusting for covariates that remained imbalanced after IPTW (Supplementary Table S26–S27).

The *E*-values calculated in the QBA analysis (Supplementary Results 1.5, Supplementary Table S28) indicated that the association of any unmeasured confounders in the study with both the treatment exposure (any characteristic that may predict or explain the difference in rate of infections) and the rate itself would need to be strong to dismiss the observed treatment effect of leniolisib on the rate of respiratory infections [43]. The *E*-values ranged from 2.99 to 5.40 (for the base case), indicating that an unmeasured confounder would need to have 5.4 times the strength of association with infection rates to be responsible for the treatment effect of leniolisib.

#### Serum IgM analysis

Baseline characteristics before and after IPTW in the base case analysis are presented in Table 3. All covariates (age at first IgM test, baseline IgM level, APDS subtype and sex) were balanced after IPTW between the treatment and control populations. Baseline characteristics before and after IPTW for the IgM sensitivity analyses (not censored for HSCT) are presented in the Supplementary Materials (Supplementary Table S29).

**Table 3:** characteristics of participants with APDS from the treatment and control groups before and after IPTW for the base case IgM analysis

	Before weighting			After weighting		
	Controls	Treatment	SMD^a^	Controls	Treatment	SMD^a^
Age at first IgM test (years)	3.0	3.3	0.059	3.3	3.3	0
Sex (% male)	54.1	49.0	0.102	56.8	56.8	0
APDS subtype (% APDS2)	18.9	28.6	0.228	23.4	23.4	0
Baseline IgM (g/L)	22.9	10.8	1.274	17.8	17.8	0

Results are reported for the base case analysis, in which missing data were handled via complete case analysis. Age at first IgM test, baseline serum IgM levels, APDS mutation status and sex were adjusted for in the propensity score model, and data were censored at first occurrence of HSCT in the outcome model. After weighting, the effective sample size was *n* = 28 in both the control and treatment group.

^a^Standardised mean difference ≥0.1 indicates imbalance.

**Abbreviations:** APDS: activated PI3K delta syndrome; ATO: average treatment effect in the overlap; HSCT: haematopoietic stem cell transplant; IgM: immunoglobulin M; IPTW: inverse probability of treatment weighting; PI3K: phosphoinositide 3-kinase; SMD: standardised mean difference.

Clinical characteristics at or prior to baseline and during follow-up for the treatment and control groups (all included participants) that were not adjusted for in the serum IgM analysis (Table 4) indicate the differences between the study group characteristics. These included increased infections at or prior to baseline in the control group, as well as malignancy, and a lower number of concomitant medications received during follow-up in the treatment group compared to the control group. Clinical characteristics that were not adjusted for in the sensitivity analysis (not censored for HSCT) are presented in the Supplementary Materials (Supplementary Table S31).

**Table 4:** clinical characteristics at or prior to baseline and during follow-up by group for the base case IgM analysis, after weighting

	At or prior to baseline			During follow-up		
	Control	Treatment	SMD^a^	Control^b^	Treatment	SMD^a^
Age at index date, years (median [IQR])	15.00 [8.00, 24.01]	16.78 [14.00, 20.00]	<0.001			
Presence of lymphoproliferation^b^ (% yes)	85.8	NA		70.1	NA	
HSCT^c^ (% yes)	0	0	<0.001	5.2	0	0.333
Infections and infestations (excluding EBV and CMV) (% yes)	98.9	47.6	1.424	100.0	65.9	1.016
Any autoimmune cytopenia (% yes)	46.9	34.5	0.255	11.8	5.8^d^	0.215
Malignancy (% yes)	17.9	3.7	0.468	14.0	3.9	0.361
Concomitant medications						
IRT (% yes)	80.1	70.5	0.224	86.6	26.9	1.510
Antibiotics (% yes)	78.6	82.1	0.088	71.4	53.5	0.376
mTOR inhibitor^e^ (% yes)	33.4	23.6	0.220	33.4	0.0	1.002
Rituximab (% yes)	5.1	0.0	0.328	5.1	0.0	0.328

^a^Standardised differences of ≥0.1 are considered meaningful.

^b^Ever-present lymphoproliferation, infections and concomitant medications are used in the absence of data variables in the ESID registry.

^c^HSCT during follow-up represents patients in the cohort who were censored during follow-up though HSCT occurred only after the observation period.

^d^Cytopenias during the leniolisib clinical studies were based on adverse event reports, and were due to any cause (so may not have been due to autoimmunity).

^e^Using definition based on any mention of mTOR throughout ESID variables.

**Abbreviations**: CMV: cytomegalovirus; EBV: Epstein-Barr virus; ESID: European Society of Immunodeficiencies; HSCT: haematopoietic stem cell transplant; IQR: interquartile range; IRT: immunoglobulin replacement therapy; mTOR: mammalian target of rapamycin; NA: not applicable; SMD: standardised mean difference.

**Figure 2:**
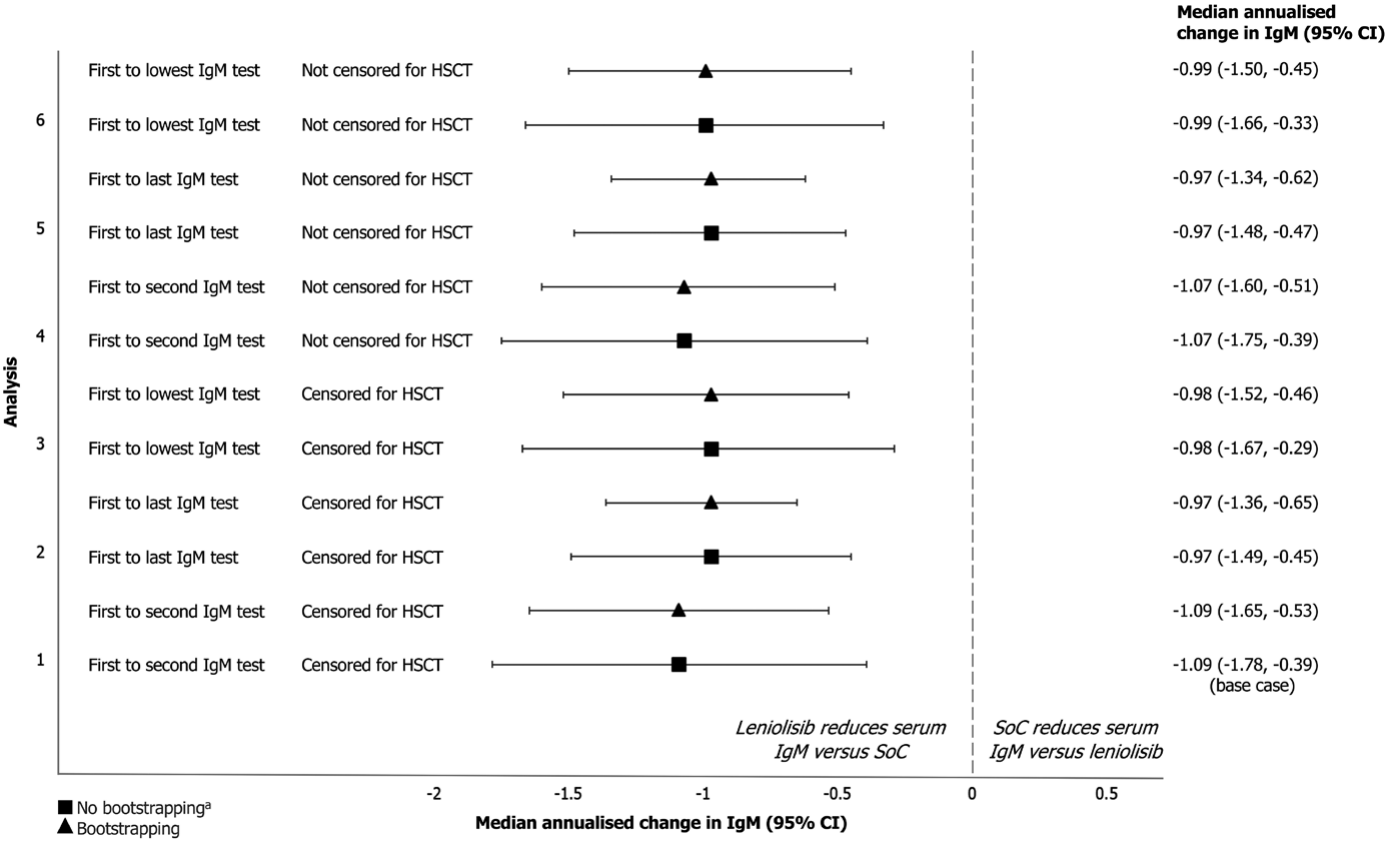
annualised change in IgM (95% CI) for the treatment versus control population across all sensitivity analyses ^a^In all analyses, 95% CIs were calculated with and without the bootstrapping method. **Abbreviations**: CI: confidence interval; HSCT: haematopoietic stem cell transplantation; IgM: immunoglobulin M; SoC: standard of care

In the base case analysis (Fig. 2), participants receiving leniolisib experienced a difference in median annualised change in IgM of −1.09 g/L (95% CI: −1.78, −0.39, *P *= 0.002) versus the control group. The observed trend was consistent when the 95% CI was calculated using the bootstrapping method, resulting in a treatment effect of −1.09 g/L (95% CI: −1.65, −0.53, *P* = 0.001) per year.

Results were consistent in the sensitivity analyses exploring the definition of annualised change in IgM (Fig. 2), using bootstrapping: comparing first to last IgM test for the control group resulted in a treatment effect of −0.97 g/L (95% CI: −1.36, −0.65, *P* = 0.001) and comparing first to lowest test for IgM for the control group resulted in a treatment effect of −0.98 g/L (95% CI: −1.52, −0.46, *P* = 0.003). Results were also consistent when censoring for HSCT was not performed (Supplementary Table S32).

## Discussion

The results of this externally controlled study, which used long-term data from Study 2201 and the ESID registry, consistently demonstrated significant reductions in the rate of respiratory tract infections, and serum IgM levels, in participants receiving leniolisib compared with participants receiving current standard of care. The observed treatment effects remained consistent regardless of missing data handling, censoring for HSCT use, definitions of baseline infection rate, definitions of annualised change in IgM and the covariates adjusted for in the propensity score model. These two endpoints were selected following feasibility assessment of the comparability and availability of data from the two data sources used. No long-term comparisons have been made between leniolisib and current standard of care for these two endpoints before.

Recurrent respiratory infections are the most commonly observed symptom in APDS (reported by between 92% and 98% of participants in a prior ESID-APDS registry analysis and a cohort study) [15, 21]. A reduction in the frequency of respiratory infections may have a positive impact on the lives of people with APDS; the symptoms of respiratory tract infections were shown to substantially reduce the HRQoL across physical, social and emotional domains in a sample of adults with upper respiratory infections in prior research [44]. Furthermore, reducing the frequency of respiratory infections may reduce the risk of developing associated organ damage, such as bronchiectasis and pulmonary fibrosis [5, 15, 23].

A decreased frequency of respiratory infections may additionally contribute to decreased dependence on treatments such as IRT, currently reported in 65–89% of people with APDS [5, 15, 23]. The potential for leniolisib to aid in reducing the treatment burden of individuals with APDS is supported by the reduction in IRT infusions and use of antibiotics observed in the OLE; 37% of patients receiving IRT at the start of the OLE experienced a reduction or discontinuation in the use of IRT [31]. Comparable data from the ESID registry, detailing IRT duration and intensity, are unfortunately limited.

Reduction in hyper-IgM values associated with APDS may be indicative of the normalisation of the disrupted processes of B cell differentiation, proliferation, maturation and function [45, 46]. In combination with the reduced incidence of respiratory infections observed here in participants treated with leniolisib, the greater reduction in serum IgM levels reported in this study supports the activity of leniolisib in enabling long-term PI3K signalling normalisation and immune system reconstitution [32]. Additionally, among the 37 patients in the leniolisib treatment arm of the OLE, three patients were long-term lymphoma survivors, as they had a history of B cell lymphoma and achieved full remission before receiving leniolisib [30, 31]; it is important to highlight the safety and efficacy of leniolisib in patients with complex medical histories, supporting its potential role in restoring immune function. Restoration of B cell function and immune reconstitution with a targeted treatment represents a key goal in the future treatment of IEI.

While this study provides novel, longer-term comparative data evidencing the potential benefit of leniolisib for reducing respiratory infections and serum IgM levels in people with APDS, there are important caveats to be considered. First, measurement bias may have been introduced by differences in the way covariates and outcomes were defined between the treatment and control groups. As data collection in the OLE study and the ESID-APDS registry were not performed under a single protocol, some of the observed treatment effects may instead be a feature of differing outcome or covariate definitions.

This study incorporated evidence from sources where pre-specified visits and data collection were not mandated, leading to missing data. Furthermore, it is anticipated that retrospective documentation of respiratory infections at yearly intervals in the ESID-APDS registry (mean number of days between visits was 387.8 days) is prone to underreporting. However, this underreporting would bias the results against leniolisib; moreover, the use of complete case and MICE analysis to handle missing data delivered consistent results with respect to both the significance and the overall magnitude of treatment effect.

As with other studies using non-randomised and indirect data to make comparisons, the possible influence of confounding should be considered. In both analyses, IPTW was used, as an established method to account for differences in the distribution of prognostically important covariates [47, 48]. Though some baseline characteristics were imbalanced post-IPTW (SMD > 0.1) for the complete case respiratory infections analysis, sensitivity analyses including these covariates in the outcomes model reached the same conclusions as the base case analysis. However, balancing was only performed for covariates deemed relevant for inclusion by clinical expert opinion; as the ESID-APDS registry was prospectively designed and APDS is a comparatively new and lesser understood condition, there may be other relevant covariates to consider.

In the respiratory infections analyses, QBA was used to assess the potential role of unmeasured confounding that had not been included in the outcome definitions. The analysis indicated that the impact of these unmeasured confounders would have to be of significant magnitude (greater than 5 times the predictive ability of the included covariates) and directionality to dismiss the observed treatment effect of leniolisib on respiratory infections. This appears unlikely, as the cohorts were generally well-balanced.

At present, the international ESID registry includes >25 000 patients with IEI, and represents the largest cohort of people with APDS globally with over 200 individuals currently enrolled [49, 50]. The registry also provides a long follow-up, with a reported median follow-up period of 6 years per patient [51]. While the generalisability of this analysis beyond Europe should be interpreted carefully, patients from the ESID-APDS registry were from a broad range of European countries, and as such, the results may be considered representative of current clinical management on a multinational scale.

## Conclusion

Overall, the magnitude and consistency of the treatment effect observed in these indirect comparisons strongly support that treatment with leniolisib reduces the rate of respiratory infections and high levels of serum IgM in people with APDS, compared with the current standard of care. This is the first study to provide longer-term comparative data on the effects of leniolisib versus standard of care on respiratory infection rates and serum IgM levels in patients with APDS. This supports that the improvements previously observed versus placebo over 12 weeks in Study 2201 are attributable to leniolisib, rather than other factors or natural history. The results demonstrate the potential for leniolisib to offer important benefits for people with APDS by reducing the high rates of infections in this population, which may avert downstream complications and the need for symptomatic treatments. Further research is required to demonstrate the clinical significance of the reduction in serum IgM levels found with leniolisib treatment and the correlation with normalisation of B cell function, which may eventually result in cessation of IRT.

## Supplementary data

Supplementary data is available at *Clinical and Experimental Immunology* online.

Collaborator Group (ESID Working Group) Members:

Additional members of the ESID Working Group who have contributed as collaborators are Stephen Jolles, Matteo Chinello, Guillaume Le Guenno, Vera Goda, Sara Kilic, Renate Krüger, Annette Uhlmann, Marina Cavazzana, Maria Fasshauer, Geraldine Blanchard-Rohner, Michaela Semeraro, Antoinette Perlat, Winnie Ip, Etienne Merlin, Jennifer Neubert, Alison Condliffe, Pere Soler-Palacin, Sylwia Koltan, Anders Fasth, Safa Baris, Pierre Frange, Antonio Marzollo, Ulrich Baumann, Saba Azarnoush, Zohreh Nademi, Bodo Grimbacher, Elif Karakoç Aydıner, Fabian Hauck, Johannes Trück, Anna Shcherbina, Marina Garcia-Prat, Hassan Abolhassani, Georgios Sogkas, Edyta Heropolitańska-Pliszka, Loic Raffray, Giorgia Bucciol, Zelimir Eric, Marketa Bloomfield, Mary Slatter, Alba Parra-Martinez, Ayca Kiykim, Nicolas Schleinitz, Jacques G. Riviere, Tomas Milota, Guillaume Lefevre, Christian Klemann, Gerhard Kindle, Evangelia Farmaki, Olov Ekwall, Lucia Pacillo, Matthew Buckland, Stephan Rusch, Alessandro Plebani, Catharina Schuetz, Vincent Barlogis, Cat Stadon, Lina Jarutyte, Seraina Prader, Benjamin Shillitoe, Nadezda Bohynikova, Carsten Speckmann, Caterina Cancrini, Beatrice Rivalta, Koen van Aerde, Nizar Mahlaoui, Federica Barzaghi, Sujal Ghosh, Peter Olbrich, Olaf Neth, Raphael Scheible and Julia Körholz.

uxae107_suppl_Supplementary_Material

## Data Availability

The following will be made available upon request: study protocol, statistical analysis plan, text, tables, figures, and appendices. Researchers who have a methodologically sound research proposal should send inquiries or requests to info@pharming.com. Data requestors may be required to sign a data access agreement.

## Abbreviations:

APDS: activated phosphoinositide 3-kinase δ syndrome
ATO: average treatment effect in the overlap
CI: confidence interval
CMV: cytomegalovirus
EBV: Epstein-Barr virus
ESID: European Society of Immunodeficiencies
HRQoL: health-related quality of life
HSCT: haematopoietic stem cell transplant
IEI: inborn error of immunity
Ig: immunoglobulin
IgA: immunoglobulin A
IgG: immunoglobulin G
IgM: immunoglobulin M
IPTW: inverse probability of treatment weighting
IQR: interquartile range
IRT: immunoglobulin replacement therapy
MICE: multiple imputation by chained equations
mTOR: mammalian target of rapamycin
NA: not applicable
OLE: open-label extension
PI3K: phosphoinositide 3-kinase
QBA: quantitative bias assessment
SMD: standardised mean difference
SoC: standard of care;
US: United States
UK: United Kingdom

## Clinical trial registration

Open-label extension study 2201E1 (NCT02859727).
