# Supplementary material for: Comparative efficacy of leniolisib (CDZ173) versus standard of care on rates of respiratory tract infection and serum immunoglobulin M (IgM) levels among individuals with activated phosphoinositide 3-kinase delta (PI3Kδ) syndrome (APDS): an externally controlled study
Source: Clin Exp Immunol. 2024 Dec 2;219(1):uxae107. doi: 10.1093/cei/uxae107 (PMC11754865; doi:10.1093/cei/uxae107)
Supplement: uxae107_suppl_Supplementary_Material [file uxae107_suppl_Supplementary_Material.docx]

Comparative efficacy of leniolisib (CDZ173) versus standard of care on rates of respiratory tract infection and serum immunoglobulin M (IgM) levels among individuals with activated phosphoinositide 3-kinase delta (PI3Kδ) syndrome (APDS): An externally-controlled study

John Whalen,^1^ Anita Chandra,^2^ Sven Kracker,^3^ Stephan Ehl,^4^ Markus G. Seidel,^5^ Ioana Gulas,^6^ Louis Dron,^6^ Russanthy Velummailum,^6^ Chenthila Nagamuthu,^6^ Sichen Liu,^6^ Joanne Tutein Nolthenius,^1^ Maria Elena Maccari,^4,7^ *on behalf of the European Society for Immunodeficiencies (ESID)-APDS Registry Working Party*

^1^Pharming Group N.V., Leiden, The Netherlands; ^2^Department of Medicine, University of Cambridge, Cambridge, UK; ^3^Université Paris Cité, Imagine Institute, Laboratory of Human Lymphohematopoiesis, INSERM UMR 1163, F-75015, Paris, France; ^4^Institute for Immunodeficiency, Center for Chronic Immunodeficiency, Medical Center-University of Freiburg, Faculty of Medicine, University of Freiburg, Freiburg, Germany; ^5^Division of Pediatric Hematology Oncology, Department of Pediatrics and Adolescent Medicine, Medical University of Graz, Austria; ^6^Cytel Inc., Toronto, Ontario, Canada; ^7^Division of Pediatric Hematology and Oncology, Department of Pediatrics and Adolescent Medicine, Medical Center-University of Freiburg, Faculty of Medicine, University of Freiburg, Freiburg, Germany

**Correspondence to:** John Whalen, J.Whalen@pharming.com

### SUPPLEMENTARY MATERIALS

Supplementary Methods 1: Statistical methods

1.1 Inverse probability of treatment weighting (IPTW)

The respiratory tract infection and the serum immunoglobulin M (IgM) analyses utilised IPTW to ensure that baseline characteristics were balanced between the treatment and control groups. Propensity scores were defined as the as the probability of a patient being assigned to leniolisib treatment conditional on the baseline covariate sets defined in each analysis.

*Respiratory infection analysis*

A logistical regression model was used to calculate propensity scores for each patient in the final respiratory infection cohort, using leniolisib treatment assignment as the dependent variable. Different combinations of the following covariates were chosen as the independent variables for each analysis:

- Age
- Sex
- APDS mutation status
- IRT use
- Baseline infection rate
- Baseline IgM

Definitions for these covariates are presented in **Table S1**.

Extreme weights were trimmed or truncated at the 5^th^ and 95^th^ percentiles, by setting weights below the 5^th^ percentile to the 5^th^ percentile, and by setting weights above the 95% percentile to the 95^th^ percentile.

*Serum IgM analysis*

A logistic regression propensity score model (PSM) was used to calculate propensity scores for each patient in the final IgM analysis cohorts. The PSM was estimated through regressing a treatment indicator variable on four baseline covariates:

- APDS mutation status
- Sex
- Age at first IgM test
- Baseline IgM (g/L)

Definitions for the baseline covariates, in addition to clinical characteristics of the treatment and control groups, are presented in **Table S2**.

Propensity scores for each patient in the cohort were calculated using fitted values from the PSM. The Average Treatment effect in the Overlap (ATO) assigned higher weights to patients with a propensity score of 0.5 (those with 50% chance of being assigned to treatment or control group), with lower scores assigned to those closer to the distribution tails (0 or 1). IPTW weights for the ATO estimand were calculated by assigning each patient the inverse of the probability of the treatment that they received.

1.2 Outcome model used in the respiratory infection analysis

The annualised rate of respiratory infections for the treatment group and the control group were modelled using a generalised linear regression model, using:

- A Conway-Maxwell Poisson distributional family
- An offset variable, to account for varying respiratory infection time intervals
- An indicator, to account for clustered patient observations

In most cases, a zero-inflation term was used to account for excess zeros. In the zero-inflation models, two models were fit:

- For the zero-component part of the model, logistic regression was used to estimate the odds ratio for not experiencing a respiratory infection
- The second component was a Conway-Maxwell distributional family count model with a log link function and an offset variable to account for the respiratory infection time intervals

To aid convergence, a mean parameterisation was applied for the Conway-Maxwell family functions.^1^ In instances where the model failed to converge, a Conway-Maxwell generalized linear model was fit or the zero-inflation term was additionally relaxed dynamically.

The conditional mean treatment effects and 95% confidence interval were then estimated. Weighted rate ratios were estimated for all analyses and weight-adjusted annualised rate of infections were estimated for the complete case analyses.

1.3 Operational definitions for covariates and clinical characteristics

Definitions for each of the covariates characterising the treatment and control groups in the respiratory infection analysis and the serum IgM analysis are described in **Table S1** and **Table S2**, respectively.

Table S1: Definitions for covariates and clinical characteristics in the respiratory infection analysis

| Characteristic | Description | Trial | ESID |
| --- | --- | --- | --- |
| Age at cohort entry | - Continuous variable, summarised by: - Median (IQR) | Age at the time of entry to the OLE study. | Year of birth was used as a proxy to calculate an estimated age by subtracting year of birth from the registry enrolment date. |
| Sex | - N (%) patients for: - Male - Female | As recorded in the dataset. | As recorded in the dataset. |
| APDS mutation type | - N (%) patients for: - APDS1 - APDS2 | As recorded in the dataset. | As recorded in the dataset. |
| Baseline infection rate (within Part I/II for the treatment population) | - Continuous variable, summarised by: - Median (IQR) | The number of infections reported (collected as AE data) in Part I/II of the trial divided by treatment time in Part I/II (84/85 says, respectively). | The number of infections reported from visit 1 to visit 2, divided by the number of days from visit 1 to visit 2. |
| Baseline infection rate (within first 183 days of OLE study for the treatment population) | - Continuous variable, summarised by: - Median (IQR) | The number of infections during the first 183 days of assessment* during the OLE study (or respective days of follow-up), divided by the follow-up time.  * If less than 183 days of study follow-up was available, the denominator was taken as the respective number of days of assessment time. | Number of infections reported from visit 1 to visit 2 divided by the number of days from visit 1 to visit 2. |
| Baseline IgM |  | The first IgM value reported during the OLE. | The IgM value recorded on or closest to the date of first visit, provided this is within one year of the visit date. |
| Baseline IRT | - N (%) patients for: - Yes | As recorded in the concomitant medication dataset. | As recorded in the dataset at first visit. |

**Abbreviations:** APDS: activated phosphoinositide 3-kinase δ syndrome; ESID: European Society for Immunodeficiencies; IgM: immunoglobulin M; IRT: immunoglobulin replacement therapy; IQR: interquartile range; N: number of patients; OLE: open-label extension.

Table S2: Definitions for covariates and clinical characteristics in the serum IgM analysis

| Variable | Description | ESID | Trial |
| --- | --- | --- | --- |
| Age at index date | - Mean (SD) - Median (IQR) - N (%) patients for: - Paediatric (<18 years) - Adult (≥18 years) - <12 years - 12–17 years - 18 years | Year of birth was used as a proxy to calculate an estimated age by subtracting year of birth from the year of IgM test date | As the majority of IgM dates were collected within the year, recorded age at baseline was used as a proxy for age at first IgM test |
| Sex | - N (%) patients for: - Male - Female | As recorded in the dataset | As recorded in the dataset |
| APDS subtype | - N (%) patients for: - APDS1 - APDS2 | As recorded in the dataset | As recorded in the dataset |
| Time from initial diagnosis to first leniolisib treatment (years) | - Mean (SD) - Median (IQR) | N/A | Calculated as difference between recorded date of diagnosis and date of first treatment dose; where inconsistencies existed between diagnosis date reported in Part I/II and the open-label extension, the earliest diagnosis date was used |
| Time from index to first leniolisib treatment (days) | - Mean (SD) - Median (IQR) | N/A | Calculated as difference between recorded index date to date of first treatment dose; most patients had first IgM test date prior to or on start of treatment, thus negative numbers were expected |
| Time from index to end of follow-up (years) | - Mean (SD) - Median (IQR) | Number of years from index to date of latest IgM sample | ADSL contains the latest treatment date per subject, used to determine end of follow-up. Number of years from index to follow-up is calculated. |
| Lymphoproliferation | - N (%) patients for: - Yes | As recorded in the dataset | All trial patients reported lymphoproliferation at baseline as per eligibility criteria for enrolment^a^ |
| Bacterial/viral/ fungal infections (excluding EBV, CMV) | - N (%) patients for: - Yes | Identified according to status recorded under the following categories: “respiratory infections”, “bacterial infections”, “acute viral infections”, “chronic viral infections”, “other infections”, “chest infections”, “other acute viral infection”, “other bacterial infection”, “viral respiratory infection”, “other infections, other” | Identified according to events recorded under “infections and infestations”, excluding EBV and CMV |
| Concomitant medications | - N (%) patients for: - Immunoglobulin replacement therapy - mTOR inhibitor - Rituximab - Antibiotics | As recorded in the dataset   - IRT: based on ‘ig_replacement’ flag in the baseline tab - mTOR inhibitors: any mention of mTOR inhibitor, rapamune, or sirolimus thoughout the dataset - Rituzimab: status under the “rituximab” variable was considered   Antibiotics: status under the “antibiotics” variable | As recorded in the dataset   - IRT: any concomitant medications categorized as “Immune Replacement Therapy” based on the ‘FDA Q Drug Classification’ variable in the ADCM dataset - mTOR inhibitors: everolimus (Afinitor, Zortress Afinitor Disperz, Votubia); sirolimus (Rapamycin, Rapamune, Fyarro); and other/unspecified mTOR inhibitors in the ADCM dataset - Rituzimab: any concomitant medications (CMTRT) labelled as ‘Rituzimab’ in the ADCM dataset. No trial patients reported Rituximab use - Antibiotics: any concomitant medications categorized as “Antibiotic” based on the ‘FDA Q Drug Classification’ variable in the ADCM dataset |
| HSCT | - N (%) patients for: - Yes - No - Missing | As recorded in the dataset | No trial patients reported HSCT at baseline |

^a^All patients were required to have lymphadenopathy as per trial eligibility criteria, however, documented clinical history of lymphoproliferation (e.g., lymphadenopathy, splenomegaly, hepatomegaly) varied. As noted in a protocol deviation, all patients except one had measurable lymphadenopathy at baseline (this patient had history of both lymphadenopathy and splenomegaly, however).

**Abbreviations:** APDS: activated PI3K delta syndrome; CMV: cytomegalovirus; EBV: Epstein-Barr virus; ESID: European Society for Immunodeficiencies; HSCT; hematopoietic stem cell transplantation; IgM: immunoglobulin M; IQR: interquartile range; mTOR: mammalian target of rapamycin; N/A: not applicable; SD: standard deviation

1.4 Transformation of annualised change in serum IgM data

The annualised change in serum IgM data was assessed for normality via a Shapiro-Wilk normality test for both the base case analysis and sensitivity analyses. The resulting distributions of annualised change in IgM for both analyses were non-normal. A power transformation of five was considered as the most appropriate for the annualised change in IgM data, as indicated by a Box-Cox power transformation applied to the cohort censored for HSCT (**Figure S1**) and not censored for HSCT (**Figure S2**).

Figure S1: Optimal power transformation for annual change in IgM (censored for HSCT)


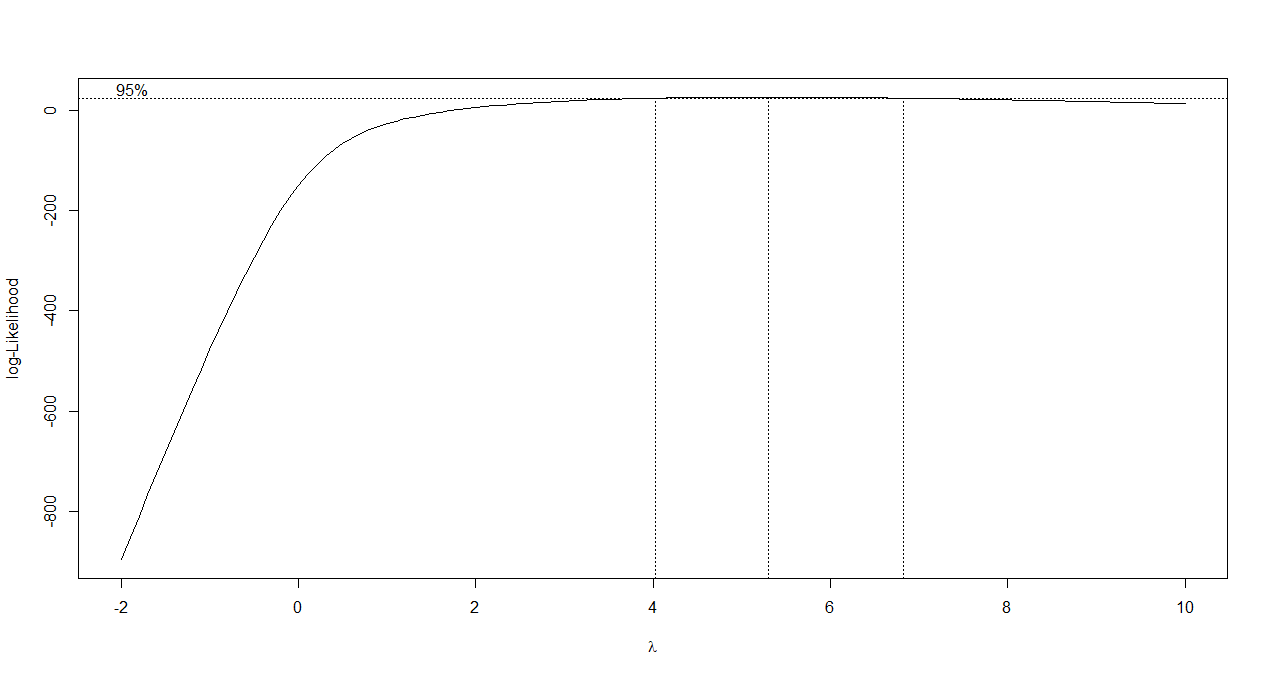


Figure S2: Optimal power transformation for annual change in IgM (not censored for HSCT)


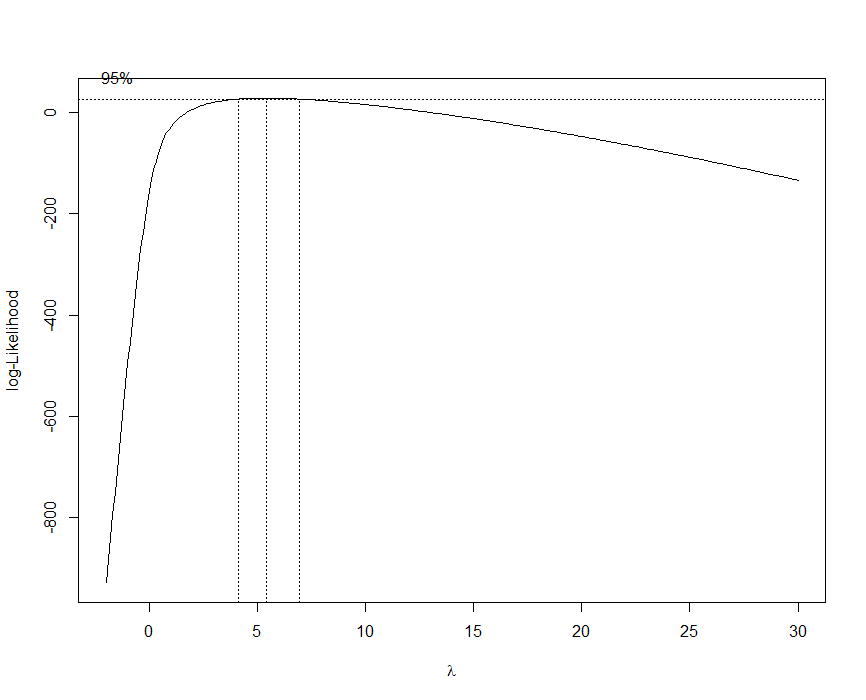


^a^These power transformations required all data to be greater than zero, thus, a linear transformation of the annualised change in IgM variable was conducted to ensure this. **Abbreviations:** IgM: Immunoglobulin M.

1.5 Multiple imputation chained equations (MICE)

For the respiratory infection analysis, regression models were constructed for each variable with missingness of data based on the conditional distributions of the other variables in the data. Imputed data sets for the patient cohorts used in the analysis (censored and not censored for HSCT) were generated via the process provided below:

1. For the variable of interest, the mean value was temporarily assigned to each of the missing values for all the variables, with missing values as a placeholder
2. The placeholder was then set back to missing for the variable of interest
3. The variable of interest was regressed on all other variables (treatment arm indicator, age at cohort entry, IgM, patient visit, baseline infection rate, exposure time of infection interval and IRT) using the most appropriate regression model, as described above. Appropriateness was chosen based on the distribution of the variable of interest
4. The missing values for the variable of interest was then replaced with predicted values estimated using the regression model constructed in the previous step. The predicted values were then subsequently used in the regression models of other variables that had missing data

The above steps were repeated for all the variables with missing data; the process of applying the above steps to all variables with missing data is defined as one cycle. At the end of one cycle, the missing values were replaced with predicted values to create a “complete” dataset. The imputation process was then repeated and generated up to 50 imputed datasets if the estimates were not stabilised; 50 imputed datasets were generated for the cohort censored at HSCT and the cohort not censored at HSCT. Exploratory analyses were performed to understand the impact of the imputation by varying this value from 30 to 500. As shown in **Table S3**, the imputation number did not demonstrate any meaningful changes to inference on increased imputations over 50.

Table S3: Summary of robustness of pooled rate ratios across 30, 50, 100 and 500 imputations

| **Analysis** | **Pooled RR across 30 Timputations** | **Pooled RR across 50 imputations** | **Pooled RR across 150 imputations** | **Pooled RR across 300 imputations** | **Pooled RR across 500 imputations** |
| --- | --- | --- | --- | --- | --- |
| **Analysis 5** | 0.377  (0.226, 0.629) | 0.377  (0.226, 0.629) | 0.377  (0.226, 0.629) | 0.377  (0.226, 0.629) | 0.377  (0.226, 0.629) |
| **Analysis 6** | 0.49  (0.299, 0.801) | 0.49  (0.299, 0.801) | 0.49  (0.299, 0.801) | 0.49  (0.299, 0.801) | 0.49  (0.299, 0.801) |
| **Analysis 7** | 0.372  (0.223, 0.621) | 0.372  (0.223, 0.621) | 0.372  (0.223, 0.621) | 0.372  (0.223, 0.621) | 0.373  (0.224, 0.623) |
| **Analysis 8** | 0.523  (0.318, 0.86) | 0.521  (0.317, 0.857) | 0.523  (0.318, 0.86) | 0.521  (0.317, 0.857) | 0.525  (0.319, 0.862) |
| **Analysis 13** | 0.417  (0.256, 0.678) | 0.418  (0.257, 0.679) | 0.417  (0.257, 0.678) | 0.418  (0.257, 0.679) | 0.418  (0.257, 0.679) |
| **Analysis 14** | 0.527  (0.324, 0.857) | 0.527  (0.324, 0.857) | 0.527  (0.324, 0.857) | 0.527  (0.324, 0.857) | 0.527  (0.324, 0.857) |
| **Analysis 15** | 0.407  (0.253, 0.656) | 0.407  (0.253, 0.657) | 0.406  (0.252, 0.655) | 0.406  (0.252, 0.654) | 0.405  (0.251, 0.655) |
| **Analysis 16** | 0.543  (0.332, 0.887) | 0.543  (0.332, 0.886) | 0.539  (0.33, 0.88) | 0.536  (0.321, 0.894) | 0.535  (0.32, 0.896) |

**Abbreviations:** HSCT: haematopoietic stem cell transplantation; RR: rate ratio.

To then estimate the rate of respiratory infections for the treatment group versus the control group, the following process was applied:

1. Weights were generated for each imputed dataset as described above in the IPTW methodology
2. The incidence rate ratio for patients in the treatment group compared to patients in the control group was estimated for each imputed dataset as described below in the outcome model methodology
3. An overall pooled estimate of the incidence rate ratio was estimated across all imputed datasets to obtain an overall incidence rate ratio
4. The standard error was then calculated, as per Rubin rules, which accounted for the variations between and within imputed datasets

1.6 Analyses explored for respiratory infection

All approaches used to determine the difference in annualised rate of respiratory infection are summarised by **Table S4**. These approaches include the handling of missing data, the covariates adjusted for, any HSCT censoring and the definition used for baseline infection rate. The base case (analysis 1) and all sensitivity analyses (analyses 2–15) for the respiratory infection analysis are therefore defined below.

Table S4: Summary of the methods used to handle missing data, the covariates adjusted in the PSM, censoring for HSCT and the definition for baseline infection rate for each analysis

| Analysis | Approach to missing data | Covariates adjusted in the propensity score model | Censored at HSCT |
| --- | --- | --- | --- |
| 1 (base case) | Complete case | Age, IRT use, baseline infection rate (within Part I/II for leniolisib arm) | Yes |
| 2 | Complete case | Age, IRT use, baseline infection rate (within first 183 days of OLE for leniolisib arm) | Yes |
| 3 | Complete case | Age, IRT use, baseline infection rate (within Part I/II for leniolisib arm), IgM, sex, APDS type | Yes |
| 4 | Complete case | Age, IRT use, baseline infection rate (within first 183 days of OLE for leniolisib arm), IgM, sex, APDS type | Yes |
| 5 | MICE | Age, IRT use, baseline infection rate (within Part I/II for leniolisib arm) | Yes |
| 6 | MICE | Age, IRT use, baseline infection rate (within first 183 days of OLE for leniolisib arm) | Yes |
| 7 | MICE | Age, IRT use, baseline infection rate (within Part I/II for leniolisib arm), IgM, sex, APDS type | Yes |
| 8 | MICE | Age, IRT use, baseline infection rate (within first 183 days of OLE for leniolisib arm), IgM, sex, APDS type | Yes |
| 9 | Complete case | Age, IRT use, baseline infection rate (within Part I/II for leniolisib arm) | No |
| 10 | Complete case | Age, IRT use, baseline infection rate (within first 183 days of OLE for leniolisib arm) | No |
| 11 | Complete case | Age, IRT use, baseline infection rate (within Part I/II for leniolisib arm), IgM, sex, APDS type | No |
| 12 | Complete case | Age, IRT use, baseline infection rate (within first 183 days of OLE for leniolisib arm), IgM, sex, APDS type | No |
| 13 | MICE | Age, IRT use, baseline infection rate (within Part I/II for leniolisib arm) | No |
| 14 | MICE | Age, IRT use, baseline infection rate (within first 183 days of OLE for leniolisib arm) | No |
| 15 | MICE | Age, IRT use, baseline infection rate (within Part I/II for leniolisib arm), IgM, sex, APDS type | No |
| 16 | MICE | Age, IRT use, baseline infection rate (within first 183 days of OLE for leniolisib arm), IgM, sex, APDS type | No |

**Abbreviations:** APDS: activated phosphoinositide 3-kinase δ syndrome; HSCT: haematopoetic stem cell transplantation; IgM: immunoglobin M; IRT: immunoglobulin replacement therapy; MICE: multiple imputation chained equations; OLE: open-label extension; PSM: propensity score model.

1.7 Analyses explored for IgM

All approaches used to determine the difference in annualised change in serum IgM are summarised in **Table S5**. Analysis 1, using the cohort censored for HSCT, forms the base case analysis of the serum IgM study. 95% confidence intervals and p-values were also calculated using the bootstrapping method (**Table S31**).

Table S5: Summary of the methods used to determine annualised change in serum IgM

| Analysis | Serum IgM tests selected for analysis (control population) | Censored at HSCT |
| --- | --- | --- |
| 1 (base case) | First to second IgM test | Yes |
| 2 | First to last IgM test | Yes |
| 3 | First to lowest IgM test | Yes |
| 4 | First to second IgM test | No |
| 5 | First to last IgM test | No |
| 6 | First to lowest IgM test | No |

**Abbreviations:** HSCT: haematopoietic stem cell transplantation; IgM: immunoglobulin M.

Supplementary Results 1: Respiratory infection analyses

1.1 Participant flow

Figure S3: Final cohort for the analysis of respiratory infection


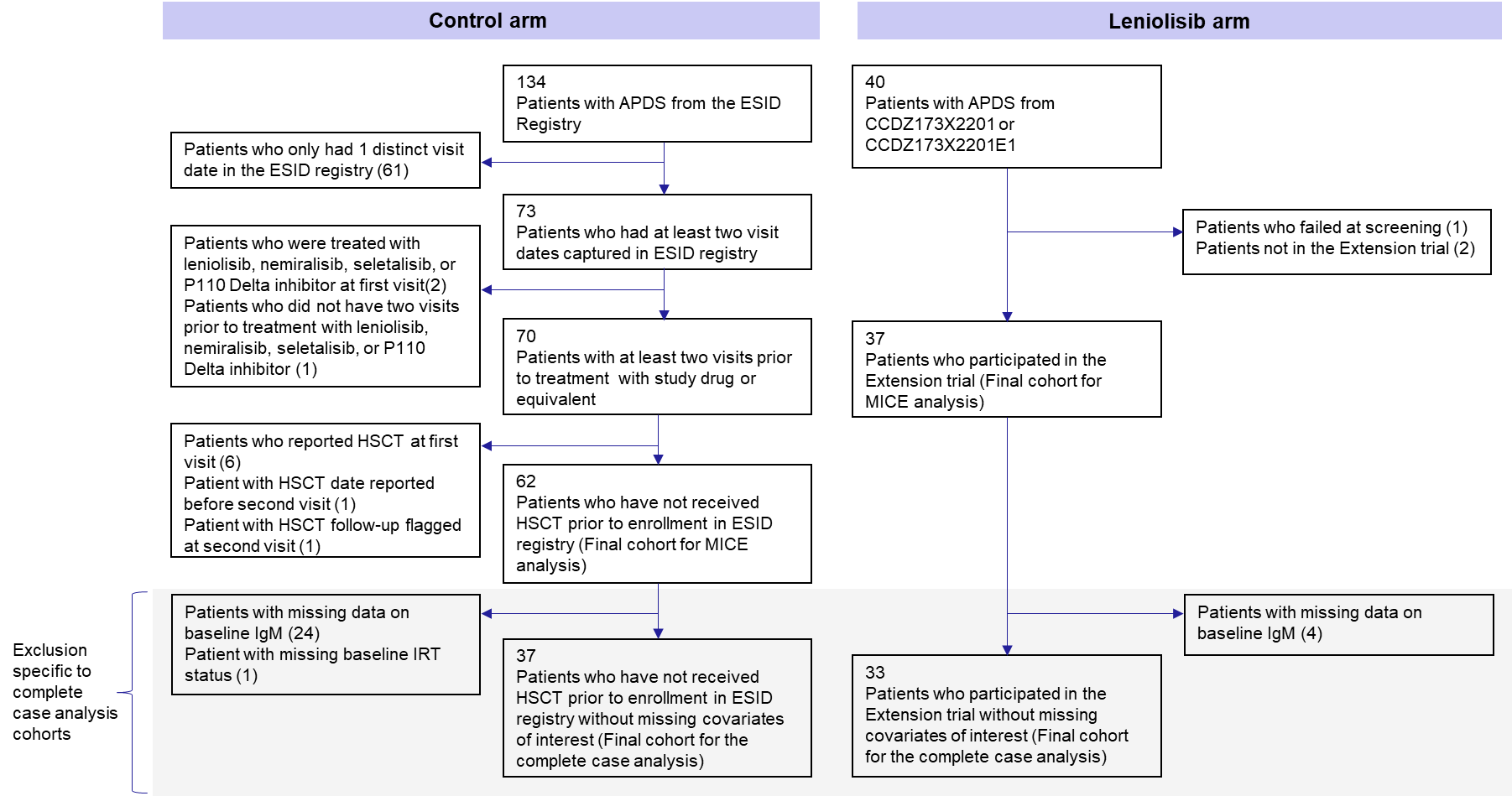


**Abbreviations:** APDS: activated phosphoinositide 3-kinase δ syndrome; ESID: European Society for Immunodeficiencies; HSCT: haematopoietic stem cell transplant; IgM: immunoglobulin M; IRT: immunoglobulin replacement therapy; MICE: multiple imputation chained equation.

1.2 Baseline and clinical characteristics of patients in the treatment and control groups for all sensitivity analyses

The following supplementary tables present the clinical characteristics of patients in the treatment and the control arms before and after IPTW based on propensity score, for all sensitivity analyses considered in the study. The baseline characteristics of patients in the cohort not censored for HSCT that were not adjusted for using IPTW in the analysis are presented in **Table S21.**

For definitions of each sensitivity analysis (numbered 2–16), see **Table S4**.

Table S6: Analysis 2 (censored at HSCT, using complete case analysis) – baseline characteristics of patients with APDS for the treatment and control arm before and after IPTW based on propensity score

| **Characteristic** | | **Before Weighting** | | | **After Weighting^a^** | | |  |
| --- | --- | --- | --- | --- | --- | --- | --- | --- |
|  |  | **Control** | **Treatment** | **SMD** | **Control** | **Treatment** | **SMD** | **Adj.^b^** |
| n | | 37 | 33 | N/A | 44.152 | 33 | N/A | N/A |
| Age at entry (median [IQR]) | | 12.000  [7.000, 21.000] | 21.000  [17.000, 29.000] | 0.624 | 27.088  [11.606, 37.252] | 20.500  [16.250, 29.000] | 0.239 | Y |
| Sex (%) | Female | 43.2 | 48.5 | 0.105 | 48.6 | 48.5 | 0.001 | N |
|  | Male | 56.8 | 51.5 |  | 51.4 | 51.5 |  |  |
| APDS (%) | APDS1 | 81.1 | 81.8 | 0.019 | 72.4 | 81.8 | 0.226 | N |
|  | APDS2 | 18.9 | 18.2 |  | 27.6 | 18.2 |  |  |
| Infection rate Part I/II, (median [IQR]) | | 0.000  [0.000, 0.006] | 0.000  [0.000, 0.012] | 0.027 | 0.000  [0.000, 0.005] | 0.000  [0.000, 0.012] | 0.293 | N |
| Infection rate, 183 days extension (median [IQR]) | | 0.000  [0.000, 0.006] | 0.000  [0.000, 0.005] | 0.469 | 0.000  [0.000, 0.005] | 0.000  [0.000, 0.005] | 0.288 | Y |
| IgM (log 10 +1) (median [IQR]) | | 0.505  [0.415, 0.598] | 0.386  [0.230, 0.659] | 0.255 | 0.494  [0.351, 0.605] | 0.385  [0.223, 0.648] | 0.052 | N |
| Baseline IRT (%) | Yes | 83.8 | 69.7 | 0.338 | 76.4 | 69.7 | 0.151 | Y |
|  | No | 16.2 | 30.3 |  | 23.6 | 30.3 |  |  |

Results are for missing data handled via complete-case analysis; age, IRT use, and baseline infection rate (within first 183 days of OLE for leniolisib arm) adjusted for in the propensity score model, and data censored at first occurrence of HSCT in the outcome model. Standardised mean difference ≥0.1 indicates imbalance. ^a^Weights were truncated at the 5^th^ and 95^th^ percentiles. ^b^Adjusted in IPTW model (Yes/No); “Y” signifies that this covariate was adjusted in the IPTW model to infer weights, while “N” signifies that covariate was not.

**Abbreviations**: Adj.: adjusted after IPTW; APDS: activated phosphoinositide 3-kinase δ syndrome; HSCT: haematopoietic stem cell transplantation; IgM: immunoglobulin M; IRT: immunoglobulin replacement therapy; IQR: interquartile range; IPTW: inverse probability of treatment weighting; N: number of patients; OLE: open-label extension; SMD: standardised mean difference.

Table S7: Analysis 3 (censored at HSCT, using complete case analysis) – baseline characteristics of patients with APDS from the treatment and control arm before and after IPTW based on propensity score

| **Characteristic** | | **Before Weighting** | | | **After Weighting^a^** | | |  |
| --- | --- | --- | --- | --- | --- | --- | --- | --- |
|  |  | **Control** | **Treatment** | **SMD** | **Control** | **Treatment** | **SMD** | **Adj.^b^** |
| n | | 37 | 33 | N/A | 43.617 | 33 | N/A | N/A |
| Age at entry (median [IQR]) | | 12.000  [7.000, 21.000] | 21.000  [17.000, 29.000] | 0.624 | 22.936  [11.289, 33.702] | 20.500  [16.250, 29.000] | 0.091 | Y |
| Sex (%) | Female | 43.2 | 48.5 | 0.105 | 48.6 | 48.5 | 0.002 | Y |
|  | Male | 56.8 | 51.5 |  | 51.4 | 51.5 |  |  |
| APDS (%) | APDS1 | 81.1 | 81.8 | 0.019 | 74.1 | 81.8 | 0.186 | Y |
|  | APDS2 | 18.9 | 18.2 |  | 25.9 | 18.2 |  |  |
| Infection rate Part I/II, (median [IQR]) | | 0.000  [0.000, 0.006] | 0.000  [0.000, 0.012] | 0.027 | 0.005  [0.000, 0.009] | 0.000  [0.000, 0.012] | 0.212 | Y |
| Infection rate, 183 days extension (median [IQR]) | | 0.000  [0.000, 0.006] | 0.000  [0.000, 0.005] | 0.469 | 0.005  [0.000, 0.009] | 0.000  [0.000, 0.005] | 0.645 | N |
| IgM (log 10 +1) (median [IQR]) | | 0.505  [0.415, 0.598] | 0.386  [0.230, 0.659] | 0.255 | 0.506  [0.389, 0.634] | 0.385  [0.223, 0.648] | 0.139 | Y |
| Baseline IRT (%) | Yes | 83.8 | 69.7 | 0.338 | 76.7 | 69.7 | 0.158 | Y |
|  | No | 16.2 | 30.3 |  | 23.3 | 30.3 |  |  |

Results are for missing data were handled via complete-case analysis; age, IRT use, baseline infection rate (within Part I/II for the leniolisib arm), IgM, sex, and APDS type adjusted for in the propensity score model, and data censored at first occurrence of HSCT in the outcome model. Standardised mean difference ≥0.1 indicates imbalance. ^a^Weights were truncated at the 5^th^ and 95^th^ percentiles. ^b^Adjusted in IPTW model (Yes/No); "Y” signifies that this covariate was adjusted in the IPTW model to infer weights, while “N” signifies that covariate was not.

**Abbreviations**: Adj.: adjusted after IPTW; APDS: activated phosphoinositide 3-kinase δ syndrome; HSCT: haematopoietic stem cell transplantation; IgM: immunoglobulin M; IRT: immunoglobulin replacement therapy; IQR: interquartile range; IPTW: inverse probability of treatment weighting; N: number of patients; OLE: open-label extension; SMD: standardised mean difference.

Table S8: Analysis 4 (censored at HSCT, using complete case analysis) – baseline characteristics of patients with APDS from the treatment and control arm before and after IPTW based on propensity score

| **Characteristic** | | **Before Weighting** | | | **After Weighting^a^** | | |  |
| --- | --- | --- | --- | --- | --- | --- | --- | --- |
|  |  | **Control** | **Treatment** | **SMD** | **Control** | **Treatment** | **SMD** | **Adj.^b^** |
| n | | 37 | 33 | N/A | 47.809 | 33 | N/A | N/A |
| Age at entry (median [IQR]) | | 12.000  [7.000, 21.000] | 21.000 [17.000, 29.000] | 0.624 | 27.407  [11.571, 38.184] | 20.500 [16.250, 29.000] | 0.243 | Y |
| Sex (%) | Female | 43.2 | 48.5 | 0.105 | 46 | 48.5 | 0.049 | Y |
|  | Male | 56.8 | 51.5 |  | 54 | 51.5 |  |  |
| APDS (%) | APDS1 | 81.1 | 81.8 | 0.019 | 75 | 81.8 | 0.167 | Y |
|  | APDS2 | 18.9 | 18.2 |  | 25 | 18.2 |  |  |
| Infection rate Part I/II, (median [IQR]) | | 0.000  [0.000, 0.006] | 0.000  [0.000, 0.012] | 0.027 | 0.000  [0.000, 0.005] | 0.000  [0.000, 0.012] | 0.316 | N |
| Infection rate, 183 days extension (median [IQR]) | | 0.000  [0.000, 0.006] | 0.000  [0.000, 0.005] | 0.469 | 0.000  [0.000, 0.005] | 0.000  [0.000, 0.005] | 0.271 | Y |
| IgM (log 10 +1) (median [IQR]) | | 0.505  [0.415, 0.598] | 0.386  [0.230, 0.659] | 0.255 | 0.486  [0.347, 0.580] | 0.385  [0.223, 0.648] | 0.012 | Y |
| Baseline IRT (%) | Yes | 83.8 | 69.7 | 0.338 | 75.9 | 69.7 | 0.139 | Y |
|  | No | 16.2 | 30.3 |  | 24.1 | 30.3 |  |  |

Results are for missing data handled via complete-case analysis, age, IRT use, baseline infection rate (within first 183 days of OLE for leniolisib arm), IgM, sex, and APDS type adjusted for in the propensity score model, and data censored at first occurrence of HSCT in the outcome model. Standardised mean difference ≥0.1 indicates imbalance. ^a^Weights were truncated at the 5^th^ and 95^th^ percentiles. ^b^Adjusted in IPTW model (Yes/No); "Y” signifies that this covariate was adjusted in the IPTW model to infer weights, while “N” signifies that covariate was not.

**Abbreviations**: Adj.: adjusted after IPTW; APDS: activated phosphoinositide 3-kinase δ syndrome; HSCT: haematopoietic stem cell transplantation; IgM: immunoglobulin M; IRT: immunoglobulin replacement therapy; IQR: interquartile range; IPTW: inverse probability of treatment weighting; N: number of patients; OLE: open-label extension; SMD: standardised mean difference.

Table S9: Analysis 5 (censored at HSCT, using MICE analysis) – baseline characteristics of patients with APDS from the treatment and control arm before and after IPTW based on propensity score

| **Characteristic** | | **Before Weighting** | | | **After Weighting^a^** | |  | |
| --- | --- | --- | --- | --- | --- | --- | --- | --- |
|  |  | **Control** | **Treatment** | **SMD** | **Control** | **Treatment** | **SMD** | **Adj.^b^** |
| Simulated n^c^ | | 3100 | 1850 | N/A | 3010 | 1850 | N/A | N/A |
| Age at entry (median [IQR]) | | 15.500 [9.000, 25.000] | 20.000 [16.000, 29.000] | 0.418 | 25.000  [15.000, 34.000] | 20.000 [16.000, 29.000] | 0.270 | Y |
| Sex (%) | Female | 48.4 | 43.2 | 0.103 | 52.2 | 43.2 | 0.180 | N |
|  | Male | 51.6 | 56.8 |  | 47.8 | 56.8 |  |  |
| APDS (%) | APDS1 | 71 | 81.1 | 0.239 | 62.1 | 81.1 | 0.431 | N |
|  | APDS2 | 29 | 18.9 |  | 37.9 | 18.9 |  |  |
| Infection rate Part I/II, (median [IQR]) | | 0.001 [0.000, 0.006] | 0.000  [0.000, 0.012] | 0.039 | 0.002  [0.000, 0.007] | 0.000 [0.000, 0.012] | 0.151 | Y |
| Infection rate, 183 days extension (median [IQR]) | | 0.001 [0.000, 0.006] | 0.000  [0.000, 0.005] | 0.487 | 0.002  [0.000, 0.007] | 0.000 [0.000, 0.005] | 0.620 | N |
| IgM (log 10 +1) (median [IQR]) | | 0.505 [0.336, 0.525] | 0.375  [0.215, 0.616] | 0.083 | 0.505  [0.193, 0.531] | 0.375 [0.215, 0.616] | 0.017 | N |
| Baseline IRT (%) | Yes | 80.5 | 67.6 | 0.299 | 69.0 | 67.6 | 0.03 | Y |
|  | No | 19.5 | 32.4 |  | 31.0 | 32.4 |  |  |

Results are for missing data handled via multiple equation chained equation; age, IRT use, and baseline infection rate (within Part I/II for the leniolisib arm) adjusted for in the propensity score model, and data censored at first occurrence of HSCT in the outcome model. Standardised mean difference ≥0.1 indicates imbalance. ^a^Weights were truncated at the 5^th^ and 95^th^ percentiles. ^b^Adjusted in IPTW model (Yes/No); "Y” signifies that this covariate was adjusted in the IPTW model to infer weights, while “N” signifies that covariate was not. ^c^While handling missing data using multiple imputation chained equation, 50 imputed datasets of Cohort 1 (censored at HSCT) before and after weighting was created.

**Abbreviations**: Adj.: adjusted after IPTW; APDS: activated phosphoinositide 3-kinase δ syndrome; HSCT: haematopoietic stem cell transplantation; IgM: immunoglobulin M; IRT: immunoglobulin replacement therapy; IQR: interquartile range; IPTW: inverse probability of treatment weighting; MICE: multiple imputation chained equation; N: number of patients; OLE: open-label extension; SMD: standardised mean difference.

Table S10: Analysis 6 (censored at HSCT, using MICE analysis) – baseline characteristics of patients with APDS from the treatment and control arm before and after IPTW based on propensity score

| **Characteristic** | | **Before Weighting** | | | **After Weighting^a^** | |  | |
| --- | --- | --- | --- | --- | --- | --- | --- | --- |
|  |  | **Control** | **Treatment** | **SMD** | **Control** | **Treatment** | **SMD** | **Adj.^b^** |
| Simulated n^c^ | | 3100 | 1850 | N/A | 2657 | 1850 | N/A | N/A |
| Age at entry (median [IQR]) | | 15.500 [9.000, 25.000] | 20.000 [16.000, 29.000] | 0.418 | 23.000 [14.000, 34.000] | 20.000  [16.000, 29.000] | 0.185 | Y |
| Sex (%) | Female | 48.4 | 43.2 | 0.103 | 50.5 | 43.2 | 0.146 | N |
|  | Male | 51.6 | 56.8 |  | 49.5 | 56.8 |  |  |
| APDS (%) | APDS1 | 71 | 81.1 | 0.239 | 65.9 | 81.1 | 0.348 | N |
|  | APDS2 | 29 | 18.9 |  | 34.1 | 18.9 |  |  |
| Infection rate Part I/II, (median [IQR]) | | 0.001  [0.000, 0.006] | 0.000  [0.000, 0.012] | 0.039 | 0.000  [0.000, 0.005] | 0.000  [0.000, 0.012] | 0.310 | N |
| Infection rate, 183 days extension (median [IQR]) | | 0.001  [0.000, 0.006] | 0.000  [0.000, 0.005] | 0.487 | 0.000  [0.000, 0.005] | 0.000  [0.000, 0.005] | 0.260 | Y |
| IgM (log 10 +1) (median [IQR]) | | 0.505  [0.336, 0.525] | 0.375  [0.215, 0.616] | 0.083 | 0.489  [0.193, 0.505] | 0.375  [0.215, 0.616] | 0.048 | N |
| Baseline IRT (%) | Yes | 80.5 | 67.6 | 0.299 | 68.4 | 67.6 | 0.018 | Y |
|  | No | 19.5 | 32.4 |  | 31.6 | 32.4 |  |  |

Results are for missing data handled via multiple equation chained equation; age, IRT use, and baseline infection rate (within first 183 days of OLE for leniolisib arm) adjusted for in the propensity score model, and data censored at first occurrence of HSCT in the outcome model. Standardised mean difference ≥0.1 indicates imbalance. ^a^Weights were truncated at the 5^th^ and 95^th^ percentiles. ^b^Adjusted in IPTW model (Yes/No); “Y” signifies that this covariate was adjusted in the IPTW model to infer weights, while “N” signifies that covariate was not. ^c^While handling missing data using multiple imputation chained equation, 50 imputed datasets of Cohort 1 (censored at HSCT) before and after weighting was created.

**Abbreviations**: Adj.: adjusted after IPTW; APDS: activated phosphoinositide 3-kinase δ syndrome; HSCT: haematopoietic stem cell transplantation; IgM: immunoglobulin M; IRT: immunoglobulin replacement therapy; IQR: interquartile range; IPTW: inverse probability of treatment weighting; MICE: multiple imputation chained equation; N: number of patients; OLE: open-label extension; SMD: standardised mean difference.

Table S11: Analysis 7 (censored at HSCT, using MICE analysis) – baseline characteristics of patients with APDS from the treatment and control arm before and after IPTW based on propensity score

| **Characteristic** | | **Before Weighting** | | | **After Weighting^a^** | |  | |
| --- | --- | --- | --- | --- | --- | --- | --- | --- |
|  |  | **Control** | **Treatment** | **SMD** | **Control** | **Treatment** | **SMD** | **Adj.^b^** |
| Simulated n^c^ | | 3100 | 1850 | N/A | 2626 | 1850 | N/A | N/A |
| Age at entry (median [IQR]) | | 15.500 [9.000, 25.000] | 20.000 [16.000, 29.000] | 0.418 | 23.000 [13.000, 30.000] | 20.000 [16.000, 29.000] | 0.106 | Y |
| Sex (%) | Female | 48.4 | 43.2 | 0.103 | 42.8 | 43.2 | 0.008 | Y |
|  | Male | 51.6 | 56.8 |  | 57.2 | 56.8 |  |  |
| APDS (%) | APDS1 | 71 | 81.1 | 0.239 | 80.4 | 81.1 | 0.017 | Y |
|  | APDS2 | 29 | 18.9 |  | 19.6 | 18.9 |  |  |
| Infection rate Part I/II, (median [IQR]) | | 0.001 [0.000, 0.006] | 0.000 [0.000, 0.012] | 0.039 | 0.002  [0.000, 0.007] | 0.000 [0.000, 0.012] | 0.107 | Y |
| Infection rate, 183 days extension (median [IQR]) | | 0.001 [0.000, 0.006] | 0.000 [0.000, 0.005] | 0.487 | 0.002 [0.000, 0.007] | 0.000 [0.000, 0.005] | 0.617 | N |
| IgM (log 10 +1) (median [IQR]) | | 0.505  [0.336, 0.525] | 0.375  [0.215, 0.616] | 0.083 | 0.505  [0.193, 0.544] | 0.375 [0.215, 0.616] | 0.079 | Y |
| Baseline IRT (%) | Yes | 80.5 | 67.6 | 0.299 | 73.6 | 67.6 | 0.132 | Y |
|  | No | 19.5 | 32.4 |  | 26.4 | 32.4 |  |  |

Results are for missing data handled via multiple equation chained equation; age, IRT use, baseline infection rate (within Part I/II for the leniolisib arm), IgM, sex, APDS mutation type adjusted for in the propensity score model, and data censored at first occurrence of HSCT in the outcome model. Standardised mean difference ≥0.1 indicates imbalance. ^a^Weights were truncated at the 5^th^ and 95^th^ percentiles. ^b^Adjusted in IPTW model (Yes/No); "Y” signifies that this covariate was adjusted in the IPTW model to infer weights, while “N” signifies that covariate was not. ^c^While handling missing data using multiple imputation chained equation, 50 imputed datasets of Cohort 1 (censored at HSCT) before and after weighting was created.

**Abbreviations**: Adj.: adjusted after IPTW; APDS: activated phosphoinositide 3-kinase δ syndrome; HSCT: haematopoietic stem cell transplantation; IgM: immunoglobulin M; IRT: immunoglobulin replacement therapy; IQR: interquartile range; IPTW: inverse probability of treatment weighting; MICE: multiple imputation chained equation; N: number of patients; OLE: open-label extension; SMD: standardised mean difference.

Table S12: Analysis 8 (censored at HSCT, using MICE analysis) – baseline characteristics of patients with APDS from the treatment and control arm before and after IPTW based on propensity score

| **Characteristic** | | **Before Weighting** | | | **After Weighting^a^** | |  | |
| --- | --- | --- | --- | --- | --- | --- | --- | --- |
|  |  | **Control** | **Treatment** | **SMD** | **Control** | **Treatment** | **SMD** | **Adj.^b^** |
| Simulated n^c^ | | 3100 | 1850 | N/A | 2732 | 1850 | N/A | N/A |
| Age at entry (median [IQR]) | | 15.500 [9.000, 25.000] | 20.000 [16.000, 29.000] | 0.418 | 22.000 [13.000, 31.000] | 20.000 [16.000, 29.000] | 0.119 | Y |
| Sex (%) | Female | 48.4 | 43.2 | 0.103 | 39.7 | 43.2 | 0.072 | Y |
|  | Male | 51.6 | 56.8 |  | 60.3 | 56.8 |  |  |
| APDS (%) | APDS1 | 71 | 81.1 | 0.239 | 82.3 | 81.1 | 0.031 | Y |
|  | APDS2 | 29 | 18.9 |  | 17.7 | 18.9 |  |  |
| Infection rate Part I/II, (median [IQR]) | | 0.001  [0.000, 0.006] | 0.000  [0.000, 0.012] | 0.039 | 0.000  [0.000, 0.005] | 0.000  [0.000, 0.0118] | 0.390 | N |
| Infection rate, 183 days extension (median [IQR]) | | 0.001  [0.000, 0.006] | 0.000  [0.000, 0.005] | 0.487 | 0.000  [0.000, 0.005] | 0.000  [0.000, 0.005] | 0.180 | Y |
| IgM (log 10 +1) (median [IQR]) | | 0.505  [0.336, 0.525] | 0.375  [0.215, 0.616] | 0.083 | 0.477  [0.193, 0.531] | 0.375  [0.215, 0.616] | 0.028 | Y |
| Baseline IRT (%) | Yes | 80.5 | 67.6 | 0.299 | 70.6 | 67.6 | 0.066 | Y |
|  | No | 19.5 | 32.4 |  | 29.4 | 32.4 |  |  |

Results are for missing data handled via multiple equation chained equation; age, IRT use, baseline infection rate (within first 183 days of OLE for leniolisib arm), IgM, sex, APDS mutation type adjusted for in the propensity score model, and data censored at first occurrence of HSCT in the outcome model. Standardised mean difference ≥0.1 indicates imbalance. ^a^Weights were truncated at the 5^th^ and 95^th^ percentiles. ^b^Adjusted in IPTW model (Yes/No); "Y” signifies that this covariate was adjusted in the IPTW model to infer weights, while “N” signifies that covariate was not. ^c^While handling missing data using multiple imputation chained equation, 50 imputed datasets of cohort 1 before and after weighting was created.

**Abbreviations**: Adj.: adjusted after IPTW; APDS: activated phosphoinositide 3-kinase δ syndrome; HSCT: haematopoietic stem cell transplantation; IgM: immunoglobulin M; IRT: immunoglobulin replacement therapy; IQR: interquartile range; IPTW: inverse probability of treatment weighting; MICE: multiple imputation chained equation; N: number of patients; OLE: open-label extension; SMD: standardised mean difference.

Clinical characteristics are presented for sensitivity analyses 9, 10, 11 and 12 (not censored for HSCT, and using complete case analysis) in Table S13, Table S14, Table S15 and Table S16, respectively.

Table S13. Analysis 9 (not censored at HSCT, using complete case analysis) – baseline characteristics of patients with APDS from the treatment and control arm before and after IPTW based on propensity score

| **Characteristic** | | **Before Weighting** | | | **After Weighting^a^** | | |  |
| --- | --- | --- | --- | --- | --- | --- | --- | --- |
|  |  | **Control** | **Treatment** | **SMD** | **Control** | **Treatment** | **SMD** | **Adj.^b^** |
| n | | 38 | 33 | N/A | 42 | 33 | N/A | N/A |
| Age at entry (median [IQR]) | | 12.500 [7.250, 21.000] | 21.000 [17.000, 29.000] | 0.628 | 25.000 [13.000, 35.797] | 20.500 [16.250, 29.000] | 0.217 | Y |
| Sex (%) | Female | 44.7 | 48.5 | 0.075 | 51.8 | 48.5 | 0.067 | N |
|  | Male | 55.3 | 51.5 |  | 48.2 | 51.5 |  |  |
| APDS (%) | APDS1 | 78.9 | 81.8 | 0.072 | 70.4 | 81.8 | 0.271 | N |
|  | APDS2 | 21.1 | 18.2 |  | 29.6 | 18.2 |  |  |
| Infection rate Part I/II, (median [IQR]) | | 0.000  [0.000, 0.006] | 0.000  [0.000, 0.012] | 0.044 | 0.002  [0.000, 0.006] | 0.000  [0.000, 0.012] | 0.078 | Y |
| Infection rate, 183 days extension (median [IQR]) | | 0.000  [0.000, 0.006] | 0.000  [0.000, 0.005] | 0.452 | 0.002  [0.000, 0.006] | 0.000  [0.000, 0.005] | 0.537 | N |
| IgM (log 10 +1) (median [IQR]) | | 0.505  [0.401, 0.595] | 0.386  [0.230, 0.659] | 0.203 | 0.505  [0.355, 0.639] | 0.385  [0.223, 0.648] | 0.090 | N |
| Baseline IRT (%) | Yes | 84.2 | 69.7 | 0.350 | 78.9 | 69.7 | 0.212 | Y |
|  | No | 15.8 | 30.3 |  | 21.1 | 30.3 |  |  |

Results are for missing data handled via complete case analysis; age, IRT use, and baseline infection rate (within Part I/II for the leniolisib arm) adjusted for in the propensity score model, and data not censored at first occurrence of HSCT in the outcome model. Standardised mean difference ≥0.1 indicates imbalance. ^a^Weights were truncated at the 5^th^ and 95^th^ percentiles. ^b^Adjusted in IPTW model (Yes/No); "Y” signifies that this covariate was adjusted in the IPTW model to infer weights, while “N” signifies that covariate was not.

**Abbreviations**: Adj.: adjusted after IPTW; APDS: activated phosphoinositide 3-kinase δ syndrome; HSCT: haematopoietic stem cell transplantation; IgM: immunoglobulin M; IRT: immunoglobulin replacement therapy; IQR: interquartile range; IPTW: inverse probability of treatment weighting; N: number of patients; OLE: open-label extension; SMD: standardised mean difference.

Table S14. Analysis 10 (not censored at HSCT, using complete case analysis) – baseline characteristics of patients with APDS from the treatment and control arm before and after IPTW based on propensity score

| **Characteristic** | | **Before Weighting** | | | **After Weighting^a^** | | |  |
| --- | --- | --- | --- | --- | --- | --- | --- | --- |
|  |  | **Control** | **Treatment** | **SMD** | **Control** | **Treatment** | **SMD** | **Adj.^b^** |
| n | | 38 | 33 | N/A | 45 | 33 | N/A | N/A |
| Age at entry (median [IQR]) | | 12.500  [7.250, 21.000] | 21.000 [17.000, 29.000] | 0.628 | 27.323  [11.852, 37.347] | 20.500  [16.250, 29.000] | 0.251 | Y |
| Sex (%) | Female | 44.7 | 48.5 | 0.075 | 49.9 | 48.5 | 0.029 | N |
|  | Male | 55.3 | 51.5 |  | 50.1 | 51.5 |  |  |
| APDS (%) | APDS1 | 78.9 | 81.8 | 0.072 | 70.3 | 81.8 | 0.273 | N |
|  | APDS2 | 21.1 | 18.2 |  | 29.7 | 18.2 |  |  |
| Infection rate Part I/II, (median [IQR]) | | 0.000  [0.000, 0.006] | 0.000  [0.000, 0.012] | 0.044 | 0.000  [0.000, 0.005] | 0.000  [0.000, 0.012] | 0.324 | N |
| Infection rate, 183 days extension (median [IQR]) | | 0.000  [0.000, 0.006] | 0.000  [0.000, 0.005] | 0.452 | 0.000  [0.000, 0.005] | 0.000  [0.000, 0.005] | 0.256 | Y |
| IgM (log 10 +1) (median [IQR]) | | 0.505  [0.401, 0.595] | 0.386  [0.230, 0.659] | 0.203 | 0.487  [0.348, 0.600] | 0.385  [0.223, 0.648] | 0.008 | N |
| Baseline IRT (%) | Yes | 84.2 | 69.7 | 0.350 | 77 | 69.7 | 0.165 | Y |
|  | No | 15.8 | 30.3 |  | 23 | 30.3 |  |  |

Results are for missing data were handled via complete case analysis; age, IRT use, and baseline infection rate (within first 183 days of OLE for leniolisib arm) adjusted for in the propensity score model, and data not censored at first occurrence of HSCT in the outcome model. Standardised mean difference ≥0.1 indicates imbalance. ^a^Weights were truncated at the 5^th^ and 95^th^ percentiles. ^b^Adjusted in IPTW model (Yes/No); "Y” signifies that this covariate was adjusted in the IPTW model to infer weights, while “N” signifies that covariate was not.

**Abbreviations**: Adj.: adjusted after IPTW; APDS: activated phosphoinositide 3-kinase δ syndrome; HSCT: haematopoietic stem cell transplantation; IgM: immunoglobulin M; IRT: immunoglobulin replacement therapy; IQR: interquartile range; IPTW: inverse probability of treatment weighting; N: number of patients; OLE: open-label extension; SMD: standardised mean difference.

Table S15. Analysis 11 (not censored at HSCT, using complete case analysis) – baseline characteristics of patients with APDS from the treatment and control arm before and after IPTW based on propensity score

| **Characteristic** | | **Before Weighting** | | | **After Weighting^a^** | | |  |
| --- | --- | --- | --- | --- | --- | --- | --- | --- |
|  |  | **Control** | **Treatment** | **SMD** | **Control** | **Tretament** | **SMD** | **Adj.^b^** |
| n | | 38 | 33 | N/A | 43 | 33 | N/A | N/A |
| Age at entry (median [IQR]) | | 12.500  [7.250, 21.000] | 21.000 [17.000, 29.000] | 0.628 | 25.000  [13.000, 34.503] | 20.500  [16.250, 29.000] | 0.190 | Y |
| Sex (%) | Female | 44.7 | 48.5 | 0.075 | 45.2 | 48.5 | 0.066 | Y |
|  | Male | 55.3 | 51.5 |  | 54.8 | 51.5 |  |  |
| APDS (%) | APDS1 | 78.9 | 81.8 | 0.072 | 77.3 | 81.8 | 0.112 | Y |
|  | APDS2 | 21.1 | 18.2 |  | 22.7 | 18.2 |  |  |
| Infection rate Part I/II, (median [IQR]) | | 0.000  [0.000, 0.006] | 0.000  [0.000, 0.012] | 0.044 | 0.003  [0.000, 0.006] | 0.000  [0.000, 0.012] | 0.007 | Y |
| Infection rate, 183 days extension (median [IQR]) | | 0.000  [0.000, 0.006] | 0.000  [0.000, 0.005] | 0.452 | 0.003  [0.000, 0.006] | 0.000  [0.000, 0.005] | 0.547 | N |
| N/A | | 0.505  [0.401, 0.595] | 0.386  [0.230, 0.659] | 0.203 | 0.523  [0.354, 0.639] | 0.385  [0.223, 0.648] | 0.109 | Y |
| Baseline IRT (%) | Yes | 84.2 | 69.7 | 0.350 | 80.2 | 69.7 | 0.244 | Y |
|  | No | 15.8 | 30.3 |  | 19.8 | 30.3 |  |  |

Results are for missing data handled via complete case analysis; age, IRT use, baseline infection rate (within Part I/II for the leniolisib arm), IgM, sex, and APDS mutation type adjusted for in the propensity score model, and data not censored at first occurrence of HSCT in the outcome model. Standardised mean difference ≥0.1 indicates imbalance. ^a^Weights were truncated at the 5^th^ and 95^th^ percentiles. ^b^ Adjusted in IPTW model (Yes/No); “Y” signifies that this covariate was adjusted in the IPTW model to infer weights, while “N” signifies that covariate was not.

**Abbreviations**: Adj.: adjusted after IPTW; APDS: activated phosphoinositide 3-kinase δ syndrome; HSCT: haematopoietic stem cell transplantation; IgM: immunoglobulin M; IRT: immunoglobulin replacement therapy; IQR: interquartile range; IPTW: inverse probability of treatment weighting; N: number of patients; OLE: open-label extension; SMD: standardised mean difference.

Table S16. Analysis 12 (not censored at HSCT, using complete case analysis) – baseline characteristics of patients with APDS from the treatment and control arm before and after IPTW based on propensity score

| **Characteristic** | | **Before Weighting** | | | **After Weighting^a^** | | |  |
| --- | --- | --- | --- | --- | --- | --- | --- | --- |
|  |  | **Control** | **Treatment** | **SMD** | **Control** | **Treatment** | **SMD** | **Adj.^b^** |
| n | | 38 | 33 | N/A | 45.874 | 33 | N/A | N/A |
| Age at entry (median [IQR]) | | 12.500  [7.250, 21.000] | 21.000 [17.000, 29.000] | 0.628 | 25.000  [11.487, 37.140] | 20.500  [16.250, 29.000] | 0.197 | Y |
| Sex (%) | Female | 44.7 | 48.5 | 0.075 | 45.3 | 48.5 | 0.064 | Y |
|  | Male | 55.3 | 51.5 |  | 54.7 | 51.5 |  |  |
| APDS (%) | APDS1 | 78.9 | 81.8 | 0.072 | 75.2 | 81.8 | 0.161 | Y |
|  | APDS2 | 21.1 | 18.2 |  | 24.8 | 18.2 |  |  |
| Infection rate Part I/II, (median [IQR]) | | 0.000  [0.000, 0.006] | 0.000  [0.000, 0.012] | 0.044 | 0.000  [0.000, 0.005] | 0.000  [0.000, 0.012] | 0.335 | N |
| Infection rate, 183 days extension (median [IQR]) | | 0.000  [0.000, 0.006] | 0.000  [0.000, 0.005] | 0.452 | 0.000  [0.000, 0.005] | 0.000  [0.000, 0.005] | 0.255 | Y |
| IgM (log 10 +1) (median [IQR]) | | 0.505  [0.401, 0.595] | 0.386  [0.230, 0.659] | 0.203 | 0.486  [0.344, 0.586] | 0.385  [0.223, 0.648] | 0.011 | Y |
| Baseline IRT (%) | Yes | 84.2 | 69.7 | 0.350 | 75.8 | 69.7 | 0.138 | Y |
|  | No | 15.8 | 30.3 |  | 24.2 | 30.3 |  |  |

Results are for missing data handled via complete case analysis; age, IRT use, baseline infection rate (within first 183 days of OLE for leniolisib arm), IgM, sex, and APDS mutation type adjusted for in the propensity score model, and data not censored at first occurrence of HSCT in the outcome model. Standardised mean difference ≥0.1 indicates imbalance. ^a^Weights were truncated at the 5th and 95th percentiles. ^b^Adjusted in IPTW model (Yes/No); "Y” signifies that this covariate was adjusted in the IPTW model to infer weights, while “N” signifies that covariate was not.

**Abbreviations**: Adj.: adjusted after IPTW; APDS: activated phosphoinositide 3-kinase δ syndrome; HSCT: haematopoietic stem cell transplantation; IgM: immunoglobulin M; IRT: immunoglobulin replacement therapy; IQR: interquartile range; IPTW: inverse probability of treatment weighting; N: number of patients; OLE: open-label extension; SMD: standardised mean difference.

Table S17. Analysis 13 (not censored at HSCT, MICE analysis) – baseline characteristics of patients with APDS from the treatment and control arm before and after IPTW based on propensity score

| **Characteristic** | | **Before Weighting** | | | **After Weighting ^a^** | |  | |
| --- | --- | --- | --- | --- | --- | --- | --- | --- |
|  |  | **Control** | **Treatment** | **SMD** | **Control** | **Treatment** | **SMD** | **Adj.^b^** |
| Simulated n^c^ | | 3200 | 1850 | N/A | 2894 | 1850 | N/A | N/A |
| Age at entry (median [IQR]) | | 16.000 [9.000, 25.000] | 20.000 [16.000, 29.000] | 0.397 | 28.000  [16.000, 34.000] | 20.000 [16.000, 29.000] | 0.313 | Y |
| Sex (%) | Female | 50 | 43.2 | 0.136 | 54.7 | 43.2 | 0.232 | N |
|  | Male | 50 | 56.8 |  | 45.3 | 56.8 |  |  |
| APDS (%) | APDS1 | 68.8 | 81.1 | 0.287 | 60.1 | 81.1 | 0.474 | N |
|  | APDS2 | 31.2 | 18.9 |  | 39.9 | 18.9 |  |  |
| Infection rate Part I/II, (median [IQR]) | | 0.001  [0.000, 0.006] | 0.000  [0.000, 0.012] | 0.048 | 0.002  [0.000, 0.006] | 0.000 [0.000, 0.012] | 0.072 | Y |
| Infection rate, 183 days extension (median [IQR]) | | 0.001  [0.000, 0.006] | 0.000  [0.000, 0.005] | 0.470 | 0.001  [0.000, 0.006] | 0.000 [0.000, 0.005] | 0.542 | N |
| IgM (log 10 +1) (median [IQR]) | | 0.438  [0.193, 0.524] | 0.375  [0.215, 0.616] | 0.020 | 0.418  [0.193, 0.507] | 0.375 [0.215, 0.616] | 0.136 | N |
| Baseline IRT (%) | Yes | 81.1 | 67.6 | 0.313 | 71.4 | 67.6 | 0.083 | Y |
|  | No | 18.9 | 32.4 |  | 28.6 | 32.4 |  |  |

Results are for missing data handled via multiple imputation chained equation; age, IRT use, and baseline infection rate (within first 183 days of OLE for leniolisib arm) adjusted for in the propensity score model, and data not censored at first occurrence of HSCT in the outcome model. Standardised mean difference ≥0.1 indicates imbalance. ^a^Weights were truncated at the 5^th^ and 95^th^ percentile. ^b^Adjusted in IPTW model (Yes/No); "Y” signifies that this covariate was adjusted in the IPTW model to infer weights, while “N” signifies that covariate was not. ^c^While handling missing data using multiple imputation chained equation, 50 imputed datasets of Cohort 1 (censored at HSCT) before and after weighting was created.

**Abbreviations**: Adj.: adjusted after IPTW; APDS: activated phosphoinositide 3-kinase δ syndrome; HSCT: haematopoietic stem cell transplantation; IgM: immunoglobulin M; IRT: immunoglobulin replacement therapy; IQR: interquartile range; IPTW: inverse probability of treatment weighting; MICE: multiple imputation chained equation; N: number of patients; OLE: open-label extension; SMD: standardised mean difference.

Table S18. Analysis 14 (not censored at HSCT, using MICE analysis) – baseline characteristics of patients with APDS from the treatment and control arm before and after IPTW based on propensity score

| **Characteristic** | | **Before Weighting** | | | **After Weighting ^a^** | |  | |
| --- | --- | --- | --- | --- | --- | --- | --- | --- |
|  |  | **Control** | **Treatment** | **SMD** | **Control** | **Treatment** | **SMD** | **Adj.^b^** |
| Simulated n^c^ | | 3200 | 1850 | N/A | 2624 | 1850 | N/A | N/A |
| Age at entry (median [IQR]) | | 16.000 [9.000, 25.000] | 20.000 [16.000, 29.000] | 0.397 | 24.000 [15.000, 34.000] | 20.000 [16.000, 29.000] | 0.217 | Y |
| Sex (%) | Female | 50 | 43.2 | 0.136 | 53.2 | 43.2 | 0.201 | N |
|  | Male | 50 | 56.8 |  | 46.8 | 56.8 |  |  |
| APDS (%) | APDS1 | 68.8 | 81.1 | 0.287 | 63 | 81.1 | 0.411 | N |
|  | APDS2 | 31.2 | 18.9 |  | 37 | 18.9 |  |  |
| Infection rate Part I/II, (median [IQR]) | | 0.001 [0.000, 0.006] | 0.000 [0.000, 0.012] | 0.048 | 0.001 [0.000, 0.005] | 0.000 [0.000, 0.012] | 0.312 | N |
| Infection rate, 183 days extension (median [IQR]) | | 0.001 [0.000, 0.006] | 0.000 [0.000, 0.005] | 0.470 | 0.001  [0.000, 0.005] | 0.000 [0.000, 0.005] | 0.246 | Y |
| IgM (log 10 +1) (median [IQR]) | | 0.438 [0.193, 0.524] | 0.375 [0.215, 0.616] | 0.020 | 0.415  [0.193, 0.505] | 0.375 [0.215, 0.616] | 0.162 | N |
| Baseline IRT (%) | Yes | 81.1 | 67.6 | 0.313 | 70.8 | 67.6 | 0.069 | Y |
|  | No | 18.9 | 32.4 |  | 29.2 | 32.4 |  |  |

Results are for missing data handled via multiple imputation chained equation; age, IRT use, and baseline infection rate (within first 183 days of OLE for leniolisib arm) adjusted for in the propensity score model, and data not censored at first occurrence of HSCT in the outcome model. Standardised mean difference ≥0.1 indicates imbalance. ^a^Weights were truncated at the 5^th^ and 95^th^ percentiles. ^b^Adjusted in IPTW model (Yes/No); “Y” signifies that this covariate was adjusted in the IPTW model to infer weights, while “N” signifies that covariate was not. ^c^While handling missing data using multiple imputation chained equation, 50 imputed datasets of Cohort 1 (censored at HSCT) before and after weighting was created.

**Abbreviations**: Adj.: adjusted after IPTW; APDS: activated phosphoinositide 3-kinase δ syndrome; HSCT: haematopoietic stem cell transplantation; IgM: immunoglobulin M; IRT: immunoglobulin replacement therapy; IQR: interquartile range; IPTW: inverse probability of treatment weighting; MICE: multiple imputation chained equation; N: number of patients; OLE: open-label extension; SMD: standardised mean difference.

Table S19. Analysis 15 (not censored at HSCT, using MICE analysis) – baseline characteristics of patients with APDS from the treatment and control arm before and after IPTW based on propensity score

| **Characteristic** | | **Before Weighting** | | | **After Weighting^a^** | |  | |
| --- | --- | --- | --- | --- | --- | --- | --- | --- |
|  |  | **Control** | **Treatment** | **SMD** | **Control** | **Treatment** | **SMD** | **Adj.^b^** |
| Simulated n^c^ | | 3200 | 1850 | N/A | 2560 | 1850 | N/A | N/A |
| Age at entry (median [IQR]) | | 16.000 [9.000, 25.000] | 20.000 [16.000, 29.000] | 0.397 | 24.000 [13.000, 31.000] | 20.000 [16.000, 29.000] | 0.146 | Y |
| Sex (%) | Female | 50 | 43.2 | 0.136 | 41.3 | 43.2 | 0.0400 | Y |
|  | Male | 50 | 56.8 |  | 58.7 | 56.8 |  |  |
| APDS (%) | APDS1 | 68.8 | 81.1 | 0.287 | 82.4 | 81.1 | 0.0350 | Y |
|  | APDS2 | 31.2 | 18.9 |  | 17.6 | 18.9 |  |  |
| Infection rate Part I/II, (median [IQR]) | | 0.001 [0.000, 0.006] | 0.000 [0.000, 0.012] | 0.0480 | 0.002 [0.000, 0.007] | 0.000 [0.000, 0.012] | 0.0110 | Y |
| Infection rate, 183 days extension (median [IQR]) | | 0.001 [0.000, 0.006] | 0.000 [0.000, 0.005] | 0.470 | 0.002  [0.000, 0.007] | 0.000 [0.000, 0.005] | 0.563 | N |
| IgM (log 10 +1) (median [IQR]) | | 0.438 [0.193, 0.524] | 0.375 [0.215, 0.616] | 0.0200 | 0.477 [0.193, 0.544] | 0.375 [0.215, 0.616] | 0.0170 | Y |
| Baseline IRT (%) | Yes | 81.1 | 67.6 | 0.313 | 75.1 | 67.6 | 0.166 | Y |
|  | No | 18.9 | 32.4 |  | 24.9 | 32.4 |  |  |

Results are for missing data handled via multiple imputation chained equation; age, IRT use, baseline infection rate (within Part I/II for the leniolisib arm), IgM, sex, and APDS mutation type adjusted for in the propensity score model, and data not censored at first occurrence of HSCT in the outcome model. Standardised mean difference ≥0.1 indicates imbalance. ^a^Weights were truncated at the 5^th^ and 95^th^ percentiles. ^b^Adjusted in IPTW model (Yes/No); “Y” signifies that this covariate was adjusted in the IPTW model to infer weights, while “N” signifies that covariate was not. ^c^While handling missing data using multiple imputation chained equation, 50 imputed datasets of Cohort 1 (censored at HSCT) before and after weighting was created.

**Abbreviations**: Adj.: adjusted after IPTW; APDS: activated phosphoinositide 3-kinase δ syndrome; HSCT: haematopoietic stem cell transplantation; IgM: immunoglobulin M; IRT: immunoglobulin replacement therapy; IQR: interquartile range; IPTW: inverse probability of treatment weighting; MICE: multiple imputation chained equation; N: number of patients; OLE: open-label extension; SMD: standardised mean difference.

Table S20. Analysis 16 (not censored at HSCT, using MICE analysis) – baseline characteristics of patients with APDS from the treatment and control arm before and after IPTW based on propensity score

| **Characteristic** | | **Before Weighting** | | | **After Weighting^a^** | |  | |
| --- | --- | --- | --- | --- | --- | --- | --- | --- |
|  |  | **Control** | **Treatment** | **SMD** | **Control** | **Treatment** | **SMD** | **Adj.^b^** |
| Simulated n^c^ | | 3200 | 1850 | N/A | 2659 | 1850 | N/A | N/A |
| Age at entry (median [IQR]) | | 16.000 [9.000, 25.000] | 20.000 [16.000, 29.000] | 0.397 | 22.000 [13.000, 31.000] | 20.000 [16.000, 29.000] | 0.115 | Y |
| Sex (%) | Female | 50 | 43.2 | 0.136 | 39.5 | 43.2 | 0.0750 | Y |
|  | Male | 50 | 56.8 |  | 60.5 | 56.8 |  |  |
| APDS (%) | APDS1 | 68.8 | 81.1 | 0.287 | 82.3 | 81.1 | 0.0310 | Y |
|  | APDS2 | 31.2 | 18.9 |  | 17.7 | 18.9 |  |  |
| Infection rate Part I/II, (median [IQR]) | | 0.001 [0.000, 0.006] | 0.000 [0.000, 0.012] | 0.0480 | 0.000  [0.000, 0.005] | 0.000 [0.000, 0.012] | 0.389 | N |
| Infection rate, 183 days extension (median [IQR]) | | 0.001 [0.000, 0.006] | 0.000 [0.000, 0.005] | 0.470 | 0.000  [0.000, 0.005] | 0.000 [0.000, 0.005] | 0.168 | Y |
| IgM (log 10 +1) (median [IQR]) | | 0.438 [0.193, 0.524] | 0.375 [0.215, 0.616] | 0.0200 | 0.415  [0.193, 0.525] | 0.375 [0.215, 0.616] | 0.124 | Y |
| Baseline IRT (%) | Yes | 81.1 | 67.6 | 0.313 | 71.3 | 67.6 | 0.0800 | Y |
|  | No | 18.9 | 32.4 |  | 28.7 | 32.4 |  |  |

Results are for missing data handled via multiple imputation chained equation; age, IRT use, baseline infection rate (within first 183 days of OLE for leniolisib arm), IgM, sex, and APDS mutation type adjusted for in the propensity score model, and data not censored at first occurrence of HSCT in the outcome model. Standardised mean difference ≥0.1 indicates imbalance. ^a^Weights were truncated at the 5^th^ and 95^th^ percentiles. ^b^Adjusted in IPTW model (Yes/No); "Y” signifies that this covariate was adjusted in the IPTW model to infer weights, while “N” signifies that covariate was not. ^c^While handling missing data using multiple imputation chained equation, 50 imputed datasets of cohort 1 before and after weighting was created.

**Abbreviations**: Adj.: adjusted after IPTW; APDS: activated phosphoinositide 3-kinase δ syndrome; HSCT: haematopoietic stem cell transplantation; IgM: immunoglobulin M; IRT: immunoglobulin replacement therapy; IQR: interquartile range; IPTW: inverse probability of treatment weighting; MICE: multiple imputation chained equation; N: number of patients; OLE: open-label extension; SMD: standardised mean difference.

Table S21: Clinical characteristics at or prior to baseline and during follow-up by group for the infections analysis (sensitivity analysis: not censored at HSCT)

|  | **At or prior to baseline** | | | **During follow-up** | | | |
| --- | --- | --- | --- | --- | --- | --- | --- |
|  | **Control** | **Treatment** | **SMD** | | **Control** | **Treatment** | **SMD** |
| N | 64 | 37 | - | | 64 | 37 | - |
| Presence of lymphoproliferation (% yes) | 87 | 100 | 0.552 | | NR | NR | - |
| HSCT (% yes)^a^ | NR | NR | - | | 15 | 0 | 0.594 |
| Respiratory tract infection (% yes)^b^ | NR | NR | - | | 73 | 62 | 0.224 |
| Concomitant medications on or prior to baseline​^a^ | | | | | | | |
| Antibiotics​ (% yes) | 48 (75) | 32 (87) | 0.136 | | 49 (77) | 24 (65) | 0.140 |
| Immunosuppressants​ | | | | | | | |
| mTOR inhibitor^c^, n (% yes) | 25 (39) | 8 (22) | 0.179 | | 31 (48) | 0 (0) | 0.696 |
| Rituximab, n​​ (% yes) | 1 (2) | 0 (0) | 0.481 | | 2 (3) | 0 (0) | 0.689 |
| Steroids^d^, n (% yes) | 30 (47) | 24 (65) | 0.274 | | 22 (34) | 23 (62) | 0.007 |

^a^Participants who received HSCT prior to or on the date of their second visit (respiratory infections analysis) or second IgM test (IgM analysis) were excluded from analysis. ^b^Dates of infection were not available for the control population, thus, 'baseline' data were not available. Baseline infection rate for the treatment population was either based on Study 2201 Part I/II (if patients were enrolled in the trial), or based on infections which occurred in the first 183 days of the OLE study to be used as a proxy rate.^c^Using definition based on any mention of mTOR throughout ESID variables. ^d^Steroid use in trial concomitant medication dataset included all drugs categorised as corticosteroids, glucocorticoids, steroid antibacterials or anticorticosteroids. **Abbreviations:** HSCT: haematopoietic stem cell transplant; mTOR: mammalian target of rapamycin; NR: not reported; SMD: standardised mean difference.

1.3 Results of the sensitivity analyses

**Table S22** and **Table S24** present results for the annualised rate of infection for the treatment and control arms, along with the respective rate ratios, for the sensitivity analyses that utilised complete case analysis for the cohort censored at HSCT and the cohort not censored at HSCT, respectively.

**Table S23** and **Table S25** present the rate ratios for the annualised rate of infections for the sensitivity analyses that utilised MICE analysis, for the cohort censored at HSCT and the cohort not censored at HSCT, respectively.

For the definitions of each sensitivity analysis presented below (numbered 2–16), see **Table S4**.

Table S22. Rate of respiratory infections in patients with APDS in the treatment arm compared to the control arm for complete case analyses 1 to 4 using the cohort censored at HSCT

| **Analysis** | **Rate ratio (95% CI)**  Weights truncated at 5^th^ and 95^th^ percentile | **Estimated annualised rate of infection for control arm** | **Estimated annualised rate of infection for treatment arm** |
| --- | --- | --- | --- |
| **1** | 0.336 (0.192, 0.590) | 1.336 (0.892, 2.002) | 0.449 (0.288, 0.700) |
| **2** | 0.405 (0.231, 0.712) | 1.128 (0.757, 1.680) | 0.457 (0.292, 0.716) |
| **3** | 0.339 (0.194, 0.594) | 1.319 (0.883, 1.970) | 0.448 (0.287, 0.697) |
| **4** | 0.420 (0.239, 0.737) | 1.086 (0.729, 1.616) | 0.456 (0.291, 0.714) |

For analyses 1-4, missing data were handled via complete case analyses and data were censored for HSCT. For analysis 1, age, IRT use, and baseline infection rate (within Part I/II for treatment arm) was adjusted for in the propensity score model. For analysis 2, age, IRT use, and baseline infection rate (within first 183 days of OLE for the treatment arm) was adjusted for in the propensity score model. For analysis 3, age, IRT use, baseline infection rate (within Part I/II for the treatment arm), IgM, sex, and APDS mutation type was adjusted for in the propensity score model. For analysis 4, age, IRT use, baseline infection rate (within first 183 days of OLE for the treatment arm), IgM, sex, and APDS mutation type was adjusted for in the propensity score model. For estimated annualised rate of infection, the rate was estimated without the zero-inflation component.

**Abbreviations**: APDS: activated phosphoinositide 3-kinase δ syndrome; CI: confidence interval; HSCT: haematopoietic stem cell transplantation; IgM: immunoglobulin replacement therapy; IRT: immunoglobulin replacement therapy; OLE: open-label extension.

Table S23. Rate of respiratory infections in patients with APDS in the treatment arm compared to the control arm for MICE analyses 5 to 8 using the cohort censored at HSCT

| **Analysis** | **Rate ratio (95% CI)**  Weights truncated at 5^th^ and 95^th^ percentile |
| --- | --- |
| **5** | 0.377 (0.226, 0.629) |
| **6** | 0.490 (0.299, 0.801) |
| **7** | 0.372 (0.223, 0.621) |
| **8** | 0.521 (0.317, 0.857) |

For analyses 5-8, missing data were handled via multiple imputation chained equation and data were censored for HSCT. For analysis 5, age, IRT use, and baseline infection rate (within Part I/II for the treatment arm) was adjusted for in the propensity score model. For analysis 6, age, IRT use, and baseline infection rate (within first 183 days of OLE for the treatment arm) was adjusted for in the propensity score model. For analysis 7, age, IRT use, baseline infection rate (within Part I/II for the treatment arm), IgM, sex, and APDS mutation type was adjusted for in the propensity score model. For analysis 8, age, IRT use, baseline infection rate (within first 183 days of OLE for the treatment arm), IgM, sex, and APDS mutation type was adjusted for in the propensity score model.

**Abbreviations**: APDS: activated phosphoinositide 3-kinase δ syndrome; CI: confidence interval; HSCT: haematopoietic stem cell transplantation; IgM: immunoglobulin M; IRT: immunoglobulin replacement therapy; MICE: multiple imputation by chained equation; OLE: open-label extension.

Table S24. Rate of respiratory infections in patients with APDS in the treatment arm compared to the control arm for compete case analyses 9 to 12 using the cohort not censored at HSCT

| **Analysis** | **Rate ratio (95% CI)**  Weights truncated at 5^th^ and 95^th^ percentile | **Estimated annualised rate of infection for control arm** | **Estimated annualised rate of infection for treatment arm** |
| --- | --- | --- | --- |
| **9** | 0.363 (0.212, 0.621) | 1.553 (0.977, 2.466) | 0.563 (0.358, 0.885) |
| **10** | 0.459 (0.263, 0.800) | 1.029 (0.691, 1.532) | 0.472 (0.303, 0.734) |
| **11** | 0.365 (0.218, 0.613) | 1.426 (0.961, 2.115) | 0.521 (0.336, 0.806) |
| **12** | 0.467 (0.270, 0.807) | 1.017 (0.686, 1.507) | 0.474 (0.307, 0.734) |

For analyses 9-12, missing data were handled via complete case analyses and data were not censored for HSCT. For analysis 9, age, IRT use, and baseline infection rate (within Part I/II for the treatment arm) was adjusted for in the propensity score model. For analysis10, age, IRT use, and baseline infection rate (within first 183 days of OLE for the treatment arm) was adjusted for in the propensity score model. For analysis 11, age, IRT use, baseline infection rate (within Part I/II for the treatment arm), IgM, sex, and APDS mutation type was adjusted for in the propensity score model. For analysis 12, age, IRT use, baseline infection rate (within first 183 days of OLE for the treatment arm), IgM, sex, and APDS mutation type was adjusted for in the propensity score model. For estimated annualised rate of infection, the rate was estimated without the zero-inflation component.

**Abbreviations**: APDS: activated phosphoinositide 3-kinase δ syndrome; CI: confidence interval; HSCT: haematopoietic stem cell transplantation; IgM: immunoglobulin replacement therapy; IRT: immunoglobulin replacement therapy; OLE: open-label extension.

Table S25. Rate of respiratory infections in patients with APDS in the treatment arm compared to the control arm for multiple imputation chained equation analyses 13 to 16 using the cohort not censored at HSCT

| **Analysis** | **Rate ratio (95% CI)**  Weights truncated at 5^th^ and 95^th^ percentile |
| --- | --- |
| **13** | 0.418 (0.257, 0.679) |
| **14** | 0.527 (0.324, 0.857) |
| **15** | 0.407 (0.253, 0.657) |
| **16** | 0.543 (0.332, 0.886) |

For analyses 13-16, missing data were handled via multiple imputation chained equation and data were not censored for HSCT. For analysis 13, age, IRT use, and baseline infection rate (within Part I/II for the treatment arm) was adjusted for in the propensity score model. For analysis14, age, IRT use, and baseline infection rate (within first 183 days of OLE for the treatment arm) was adjusted for in the propensity score model. For analysis 15, age, IRT use, baseline infection rate (within Part I/II for the treatment arm), IgM, sex, and APDS mutation type was adjusted for in the propensity score model. For analysis 16, age, IRT use, baseline infection rate (within first 183 days of OLE for the treatment arm), IgM, sex, and APDS mutation type was adjusted for in the propensity score model.

**Abbreviations**: APDS: activated phosphoinositide 3-kinase δ syndrome; CI: confidence interval; HSCT: haematopoietic stem cell transplantation; IgM: immunoglobulin M; IRT: immunoglobulin replacement therapy; MICE: multiple imputation by chained equation; OLE: open-label extension.

1.4 Adjusted rate ratios

**Table S26** and **Table S27** present the resulting rate ratios after adjustment of the remaining unbalanced covariates after IPTW in the outcome model.

**Table S26** presents analyses 3, 4, 5 and 6 relevant to the cohort censored at HSCT, while **Table S27** presents analyses 11, 12, 15 and 16 relevant to the cohort not censored at HSCT. In these tables, analyses denoted ‘.1’ indicate the results for the analysis after adjustment of the residual unbalanced covariates after IPTW.

Table S26: Comparison of rate ratios that were unadjusted vs. adjusted for imbalanced covariates in the outcome model for Cohort 1 (not censored at HSCT)

| **Analysis** | **Rate ratio (95% CI)^a^** | **Imbalance adjusted analysis** | **Adjusted rate ratio ^a^ (95% CI)** | **Imbalanced covariates adjusted** |
| --- | --- | --- | --- | --- |
| **3** | 0.339 (0.194, 0.594) | **3.1** | 0.362 (0.207, 0.634) | APDS, IgM (log 10 +1), Baseline IRT |
| **4** | 0.420 (0.239, 0.737) | **4.1** | 0.410 (0.232, 0.722) | Age at entry, APDS, Baseline IRT |
| **7** | 0.372 (0.223, 0.621) | **7.1** | 0.381 (0.229, 0.636) | Age at entry, Baseline IRT |
| **8** | 0.521 (0.317, 0.857) | **8.1** | 0.522 (0.319, 0.854) | Age at entry |

^a^Estimated weights are truncated at the 5^th^ and 95^th^ percentile.

**Abbreviations:** APDS: activated phosphoinositide 3-kinase δ syndrome; CI: confidence interval; HSCT: haematopoetic stem cell transplantation; IgM: immunoglobulin M; IRT: immunoglobulin replacement therapy.

Table S27: Rate ratios compared with rate ratios adjusted by imbalanced covariates in the outcome model for Cohort 2 (not censored at HSCT)

| **Analysis** | **Rate ratio (95% CI)^a^** | **Imbalance adjusted analysis** | **Adjusted rate ratio^a^ (95% CI)** | **Imbalanced covariates adjusted** |
| --- | --- | --- | --- | --- |
| **11** | 0.365 (0.218, 0.613) | **11.1** | 0.372 (0.22, 0.628) | Age at entry, APDS, Baseline IRT |
| **12** | 0.467 (0.27, 0.807) | **12.1** | 0.445 (0.26, 0.764) | Age at entry, APDS, Baseline IRT |
| **15** | 0.407 (0.253, 0.657) | **15.1** | 0.42 (0.265, 0.667) | Age at entry, Baseline IRT |
| **16** | 0.543 (0.332, 0.886) | **16.1** | 0.557 (0.343, 0.903) | Age at entry, IgM (log 10 +1) |

^a^Estimated weights are truncated at the 5^th^ and 95^th^ percentiles.

**Abbreviations:** APDS: activated phosphoinositide 3-kinase δ syndrome; CI: confidence interval; HSCT: haematopoetic stem cell transplantation; IgM: immunoglobulin M; IRT: immunoglobulin replacement therapy.

1.5 E-values generated for the respiratory infection analyses

E values were calculated for all analyses performed, including those analyses adjusted for imbalanced covariates in the outcome model (as denoted by ‘.1’ for each relevant analysis).^2, 3^ These E values were calculated with respect to the rate of respiratory infections between treatment arms and are presented in Table S28.

Table S28: Rate ratios and observed covariate E-values for all analyses

| **Analysis** | **Rate ratio ^a^ (95% CI)** | **E-value** | **Analysis** | **Rate ratio ^a^ (95% CI)** | **E-value** |
| --- | --- | --- | --- | --- | --- |
| **1** | 0.336 (0.192, 0.590) | 5.401 | **9** | 0.363 (0.212, 0.621) | 4.954 |
| **2** | 0.405 (0.231, 0.712) | 4.374 | **10** | 0.459 (0.263, 0.800) | 3.781 |
| **3** | 0.339 (0.194, 0.594) | 5.348 | **11** | 0.365 (0.218, 0.613) | 4.923 |
| **3.1^b^** | 0.362 (0.207, 0.634) | 4.969 | **11.1^b^** | 0.372 (0.220, 0.628) | 4.818 |
| **4** | 0.420 (0.239, 0.737) | 4.194 | **12** | 0.467 (0.270, 0.807) | 3.705 |
| **4.1^b^** | 0.410 (0.232, 0.722) | 4.312 | **12.1^b^** | 0.445 (0.260, 0.764) | 3.921 |
| **5** | 0.377 (0.226, 0.629) | 4.746 | **13** | 0.418 (0.257, 0.679) | 4.217 |
| **6** | 0.490 (0.299, 0.801) | 3.498 | **14** | 0.527 (0.324, 0.857) | 3.203 |
| **7** | 0.372 (0.223, 0.621) | 4.818 | **15** | 0.407 (0.253, 0.657) | 4.349 |
| **7.1^b^** | 0.381 (0.229, 0.636) | 4.690 | **15.1^b^** | 0.420 (0.265, 0.667) | 4.194 |
| **8** | 0.521 (0.317, 0.857) | 3.248 | **16** | 0.543 (0.332, 0.886) | 3.087 |
| **8.1^b^** | 0.522 (0.319, 0.854) | 3.240 | **16.1^b^** | 0.557 (0.343, 0.903) | 2.990 |

^a^Estimated weights are truncated at the 5^th^ and 95^th^ percentiles.

^b^Analyses were adjusted by imbalanced covariates.

**Abbreviations:** CI: confidence interval.

Supplementary Results 2: Serum IgM analysis

2.1 Participant flow

Figure S4: Flow diagram illustrating cohort creation for the IgM analysis (censored for HSCT)


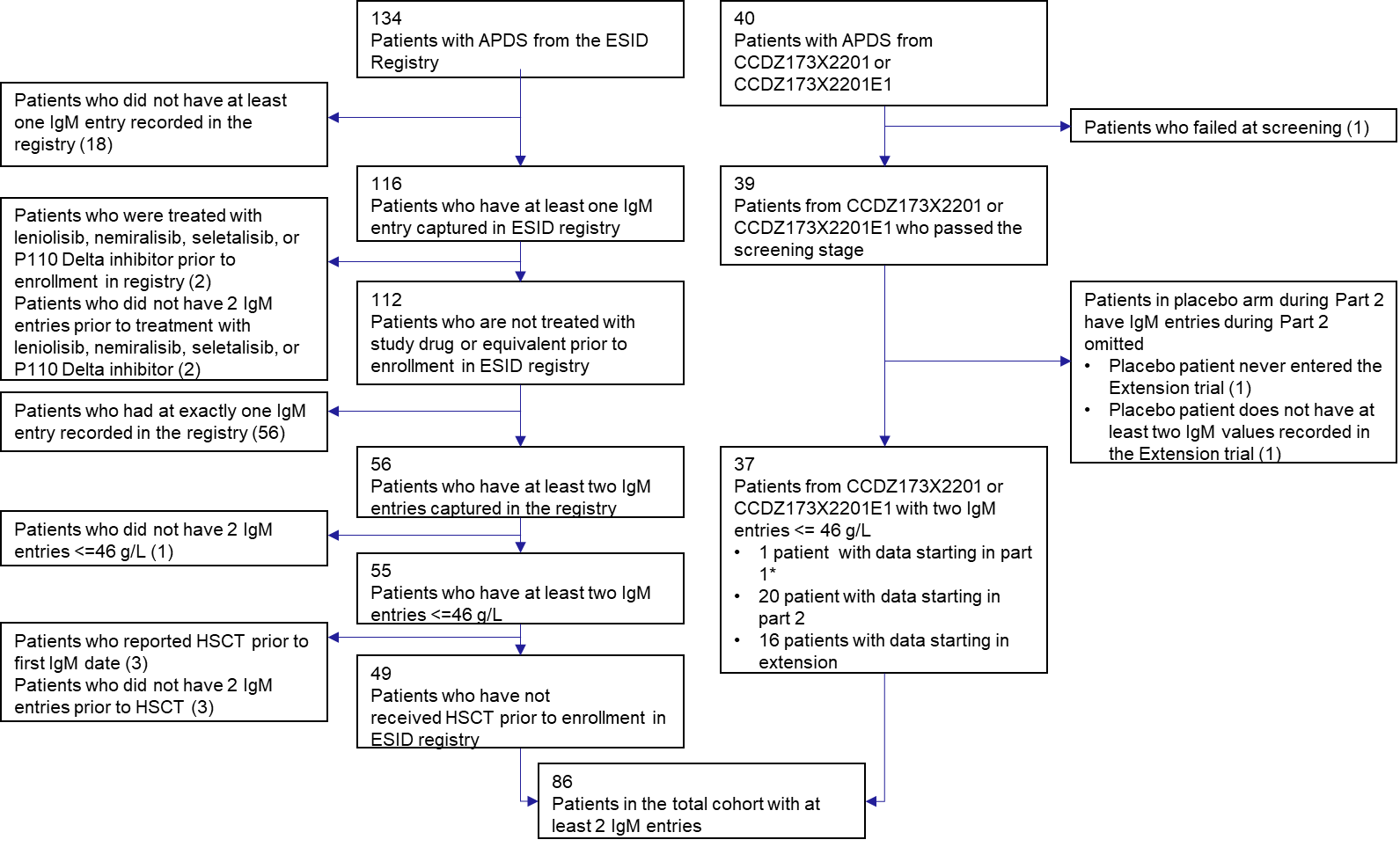


^*^ The remaining five patients from Part I had a >6-week treatment-free period between Part I and the OLE. For this analysis, baseline was defined as the first visit in the OLE.

**Abbreviations:** APDS: activated PI3K delta syndrome; ESID: European Society for Immunodeficiency; HSCT: haematopoetic stem cell transplantation; IgM: immunoglobulin M.

Figure S5: Flow diagram illustrating cohort creation for the IgM analysis (not censored for HSCT)


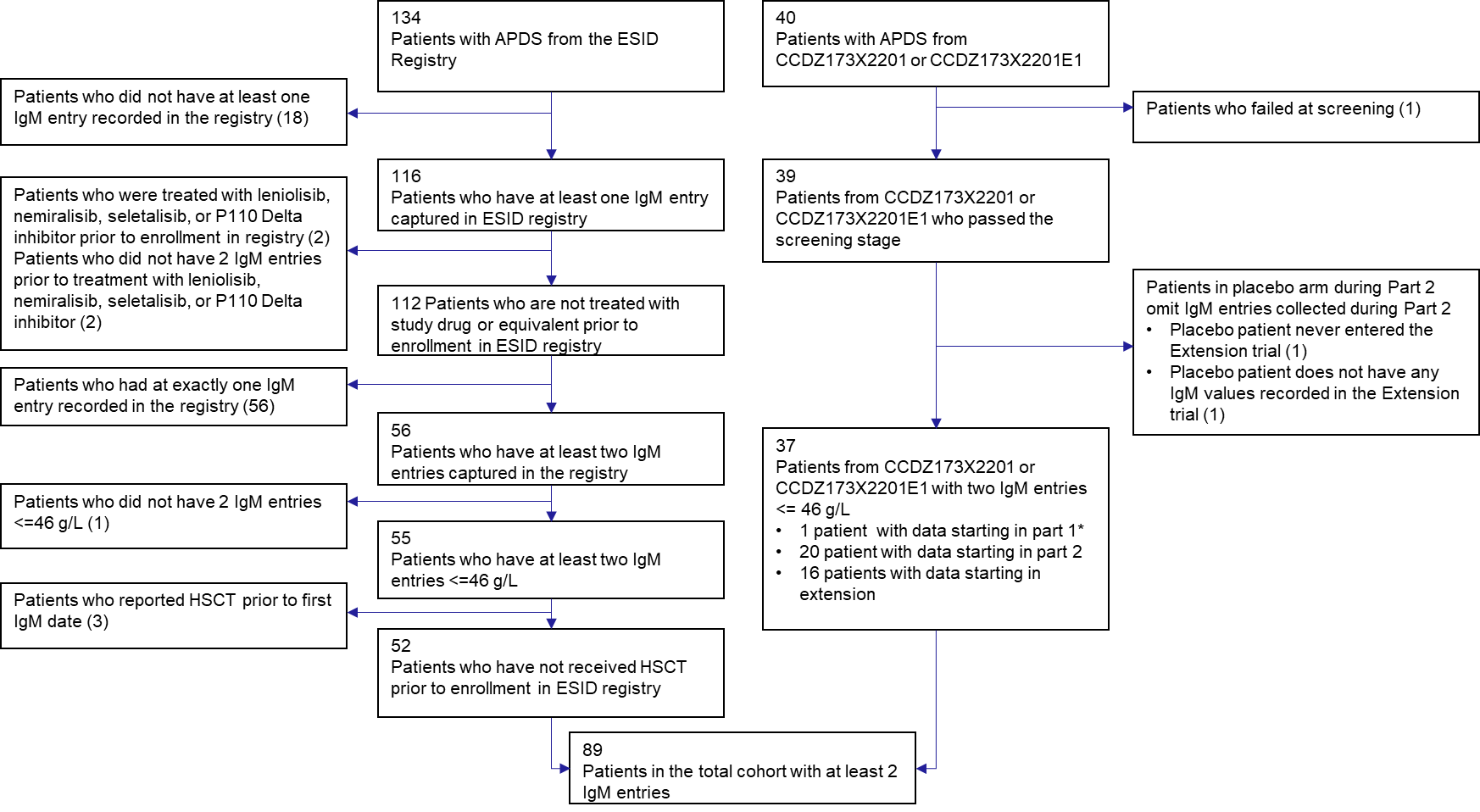


^*^ The remaining five patients from Part I had a >6-week treatment-free period between Part I and the OLE. For this analysis, baseline was defined as the first visit in the OLE.

**Abbreviations:** APDS: activated PI3K delta syndrome; ESID: European Society for Immunodeficiency; HSCT: haematopoetic stem cell transplantation; IgM: immunoglobulin M.

2.2 Results of the sensitivity analysis

The final cohort for the sensitivity analysis of IgM (not censored for HSCT) consisted of 37 treated and 52 control participants. Unadjusted clinical characteristics at baseline and during follow-up for the treatment and control groups are presented in **Table S29**.

Table S29: Analyses 4–6 (not censored for HSCT) – Clinical characteristics at or prior to baseline and during follow-up by group (all included participants)

|  | **At or prior to baseline** | | | **During follow-up** | | |
| --- | --- | --- | --- | --- | --- | --- |
|  | **Control** | **Treatment** | **Standardised difference^a^** | **Control^b^** | **Treatment** | **Standardised difference^a^** |
| N | 52 | 37 | N/A | 52 | 37 | N/A |
| Age at index date (years), mean ± SD | 10.67 ± 8.73 | 22.86 ± 10.02 | - | N/A | N/A | N/A |
| Presence of lympho-proliferation, n (%) | 43 (82.7) | 36 (97.3) | 0.283 | 35 (67.3) | 37 (100) | 0.410 |
| Infections and infestations (excluding EBV and CMV), n (%) | 50 (96) | 21 (57) | 0.483 | 46 (88) | 30 (81) | 0.103 |
| Concomitant medications on or prior to baseline​ | | | | | | |
| IRT | 41 (79) | 26 (70) | 0.098 | 46 (88) | 13 (35) | 0.556 |
| Antibiotics​ (% yes) | 39 (75) | 29 (78) | 0.039 | 41 (79) | 20 (54) | 0.282 |
| mTOR inhibitor^c^​, n (% yes) | 23 (44) | 7 (19) | 0.394 | 27 (52) | 0 (0) | 0.720 |
| Rituximab​ ​ (% yes) | 1 (2) | 0 (0) | 0.481 | 1 (1.9) | 0 (0) | 0.562 |

^a^Standardised differences of ≥0.1 are considered meaningful. ^b^Ever-present lymphoproliferation, infections and concomitant medications are used in the absence of data variables in the ESID registry. ^c^ Using definition based on any mention of mTOR throughout ESID variables.

**Abbreviations:** CMV: cytomegalovirus; EBV: Epstein-Barr virus; ESID: European Society for Immunodeficiencies; HSCT: haematopoietic stem cell transplant; IRT: immunoglobulin replacement therapy; mTOR: mammalian target of rapamycin; SD: standard deviation.

A summary of covariant balance achieved via propensity scoring is provided in **Table S30**. A summary of the resulting effective cohort sample size, and adjusted sample sizes of the treatment and control group, are presented in **Table S31**.

Table S30: Analyses 4–6 (not censored for HSCT) – IPTW to estimate ATO, modelling annualised change in IgM as outcome

|  | **Before Weighting** | | | **After Weighting** | | |
| --- | --- | --- | --- | --- | --- | --- |
|  | **Treatment** | **Control** | **SMD** | **Treatment** | **Control** | **SMD** |
| Propensity score | 0.270 | 0.621 | 1.497 | 0.482 | 0.489 | 0.029 |
| Age at 1st IgM test (years) | 3.178 | 3.050 | 0.036 | 3.282 | 3.282 | 0 |
| Baseline IgM (g/L) | 10.673 | 22.865 | 1.297 | 17.789 | 17.789 | 0 |
| APDS subtype (% APDS2) | 0.289 | 0.189 | 0.234 | 0.238 | 0.238 | 0 |
| Sex (% male) | 0.481 | 0.541 | 0.120 | 0.564 | 0.564 | 0 |

**Abbreviations:** APDS: activated PI3K delta syndrome; ATO: average treatment effect in the overlap; HSCT: haematopoietic stem cell transplant; IgM: immunoglobulin M; IPTW: inverse probability of treatment weighting; SMD: standardised mean difference.

Table S31: Analyses 4–6 (not censored for HSCT) – Effective sample size

| **Analysis** | **Effective cohort sample size** | **Adjusted sample size of treatment group** | **Adjusted sample size of control group** |
| --- | --- | --- | --- |
| **Not censored for HSCT** | 56.59 | 27.48 | 29.11 |

Results in the difference in change in annualised IgM remained consistent in all sensitivity analyses, including those with confidence intervals calculated using the bootstrapping method, with respect to the base case IgM analysis, as shown by **Table S32.**

Table S32: Median annualised change in IgM for analyses 2–6 in the IgM analysis

| Analysis | Median annualised change in IgM | 95% CI | p-value |
| --- | --- | --- | --- |
| 2: First to last IgM test, censored for HSCT | -0.97 | -1.49, -0.45 | 0.0002 |
| 2: First to last IgM test, censored for HSCT (bootstrapping method) | -0.97 | -1.36, -0.65 | 0.0010 |
| 3: First to lowest IgM test, censored for HSCT | -0.98 | -1.67, -0.29 | 0.0055 |
| 3: First to lowest IgM test, censored for HSCT (bootstrapping method) | -0.98 | -1.52, -0.46 | 0.0030 |
| 4: First to second IgM test, not censored for HSCT | -1.07 | -1.75, -0.39 | 0.0020 |
| 4: First to second IgM test, not censored for HSCT (bootstrapping method) | -1.07 | -1.60, -0.51 | 0.0040 |
| 5: First to last IgM test, not censored for HSCT | -0.97 | -1.48, -0.47 | 0.0002 |
| 5: First to last IgM test, not censored for HSCT (bootstrapping method) | -0.97 | -1.34, -0.62 | 0.0010 |
| 6: First to lowest IgM test, not censored for HSCT | -0.99 | -1.66, -0.33 | 0.0035 |
| 6: First to lowest IgM test, not censored for HSCT (bootstrapping method) | -0.99 | -1.50, -0.45 | 0.0040 |

**Abbreviations:** CI: confidence interval; HSCT: haematopoetic stem cell transplantation; IgM: Immunoglobulin M.

### References

1. Huang A. Mean-parametrized Conway–Maxwell–Poisson regression models for dispersed counts. Statistical Modelling 2017;17:359-380.

2. VanderWeele TJ, Ding P. Sensitivity analysis in observational research: introducing the E-value. Annals of Internal Medicine 2017;167:268-74.

3. Ding P, VanderWeele TJ. Sensitivity Analysis Without Assumptions. Epidemiology 2016;27:368-377.
